# Modeling Selenoprotein *Se*-Nitrosation: Synthesis of a *Se*-Nitrososelenocysteine with Persistent Stability

**DOI:** 10.1021/jacs.3c03394

**Published:** 2023-06-02

**Authors:** Ryosuke Masuda, Satoru Kuwano, Kei Goto

**Affiliations:** Department of Chemistry, School of Science, Tokyo Institute of Technology, 2-12-1 Ookayama, Meguro-ku, Tokyo 152-8551, Japan

## Abstract

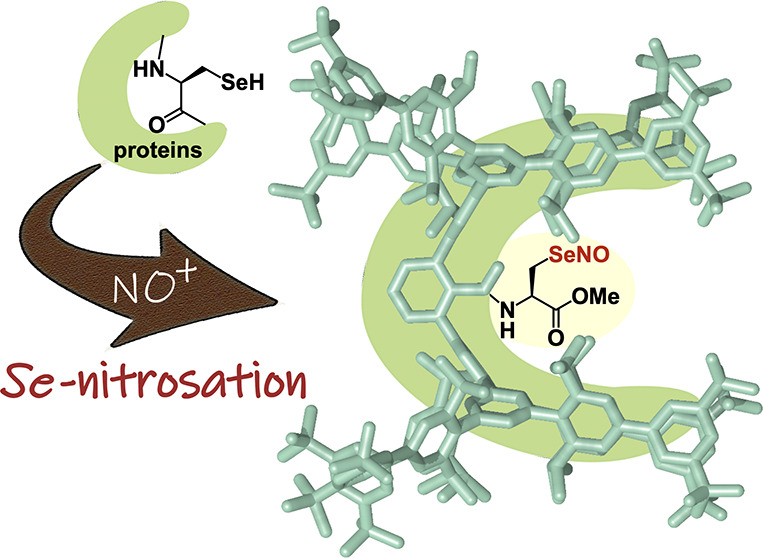

The *Se*-nitrosation in selenoproteins such as glutathione peroxidase and thioredoxin reductase to produce *Se*-nitrososelenocysteines (Sec–SeNOs) has been proposed to play crucial roles in signaling processes mediated by reactive nitrogen species and nitrosative-stress responses, although chemical evidence for the formation of Sec–SeNOs has been elusive not only in proteins but also in small-molecule systems. Herein, we report the first synthesis of a Sec–SeNO by employing a selenocysteine model system that bears a protective molecular cradle. The Sec–SeNO was characterized using ^1^H and ^77^Se nuclear magnetic resonance as well as ultraviolet/visible spectroscopy and found to have persistent stability at room temperature in solution. The reaction processes involving the Sec–SeNO provide experimental information that serves as a chemical basis for elucidating the reaction mechanisms involving the SeNO species in biological functions, as well as in selenol-catalyzed NO generation from *S*-nitrosothiols.

Glutathione peroxidase (GPx)^1^ and thioredoxin reductase (TrxR)^2^ are selenoenzymes that play important and well-established roles in signaling processes mediated by reactive oxygen species (ROS) and oxidative-stress responses.^3,4^ Since the 1990s, an increasing amount of research has indicated that GPx and TrxR are also crucial in signaling processes mediated by reactive nitrogen species (RNS) and nitrosative-stress responses.^5−8^ In particular, GPx and TrxR constitute key components in cellular redox pathways involved in the metabolism of *S*-nitrosothiols (RSNOs) and peroxynitrite; by promoting the metabolism of RSNOs and peroxynitrite, GPx and TrxR are integrated into the mammalian nitrosative-stress response.^6c,8,9^ It has also been reported that GPx and TrxR are sensitive to RNS and are inactivated by RSNOs and peroxynitrite. In the interactions between these selenoenzymes and RNS, the SeH groups of the selenocysteine (Sec) residues in their active sites are critically involved.^5b^ Based on the inherent high nucleophilicity of selenols, the *Se*-nitrosation of Sec–SeH to produce *Se*-nitrososelenoycsteines (Sec–SeNOs) is expected in RNS-treated selenoproteins (Figure 1a),^6b,10^ just as the oxidation of Sec–SeHs with ROS produces selenocysteine selenenic acids (Sec–SeOHs) as reactive intermediates.^11^ However, in sharp contrast to the ubiquitous *S*-nitrosation^12^ of various proteins and peptides,^13^ SeNO modification has not yet been identified in any selenoproteins. Moreover, the observation of a Sec–SeNO in a small-molecule system has not been reported so far. Yet, for the identification of the *Se*-nitrosated forms of selenoproteins and the chemical elucidation of their roles in RNS-mediated signaling processes and nitrosative-stress responses, the development of a small-molecule model compound for a Sec–SeNO with sufficient stability is highly desirable.

**Figure 1:**
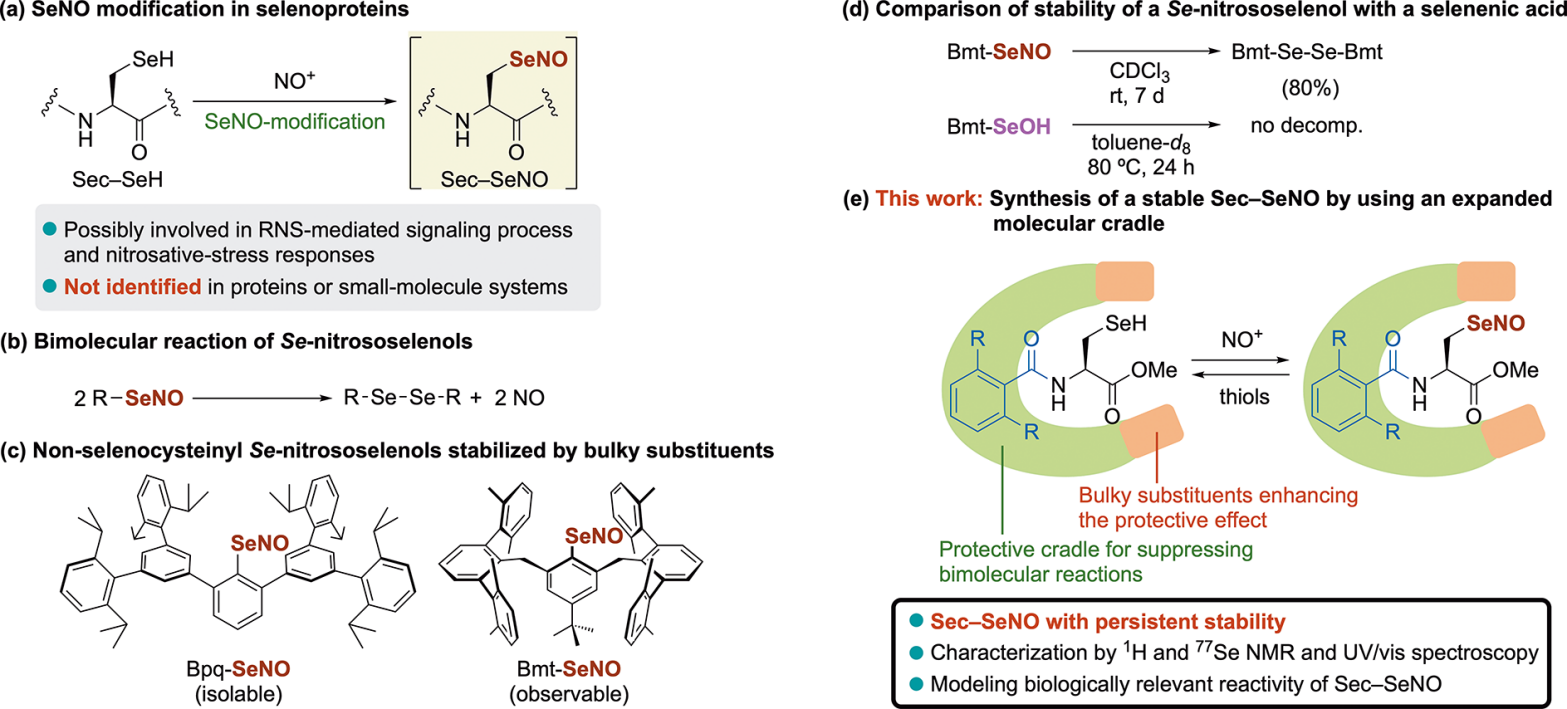
Previous works on *Se*-nitrososelenols and conceptual illustration of this study.

The formation of *Se*-nitrososelenols for nonselenocysteinyl derivatives has been experimentally demonstrated by our group and others. We have already reported the synthesis of BpqSeNO^14^ and BmtSeNO^15^ as stable *Se*-nitrososelenols with bulky aromatic substituents (Figure 1c). Moreover, du Mont and co-workers have reported the generation of (Me_3_Si)_3_CSeNO, which was suggested by IR spectroscopy at −78 °C, although it decomposed upon warming.^16^ Mardyukov and co-workers have reported the spectroscopic identification of MeSeNO in an argon matrix at 10 K.^17^

The studies of these nonselenocysteinyl derivatives revealed that *Se*-nitrososelenols are more prone to bimolecular decomposition (Figure 1b) involving the formation of Se–Se bonds than other selenium-containing reactive species such as selenenic acids, which are notoriously labile due to facile self-condensation.^18^ We found that BmtSeNO gradually decomposes to the corresponding diselenide at room temperature in solution,^15^ while the selenenic acid bearing the same substituent, BmtSeOH,^19^ exhibited high stability upon heating (Figure 1d). This instability of nonselenocysteinyl *Se*-nitrososelenols suggests that stabilizing Sec–SeNOs in small-molecule systems would be extremely difficult.

During our studies on modeling the reactive intermediates in the catalytic cycle of selenoenzymes, we have recently succeeded in the first observation of a Sec–SeOH^20,21^ by employing a large molecular cradle^22^ as an *N*-terminal protecting group (henceforth denoted as “Bpsc”; Figure 2A). To overcome the more facile bimolecular decomposition of Sec–SeNOs, we designed an expanded molecular cradle with peripheral 3,5-di-*tert*-butylphenyl units (henceforth denoted as “DB-Bpsc”; Figure 2B). Herein, we report the first synthesis of a Sec–SeNO, which is formed by *Se*-nitrosation of a selenocysteine model compound that bears the expanded molecular cradle (Figure 1e). The small-molecule Sec–SeNO, which was characterized by ^1^H and ^77^Se nuclear magnetic resonance (NMR) spectroscopy as well as ultraviolet–visible (UV–vis) spectroscopy, exhibited persistent stability at room temperature in solution. Some biologically relevant reaction processes involving Sec–SeNOs were investigated using this stable model compound.

**Figure 2:**
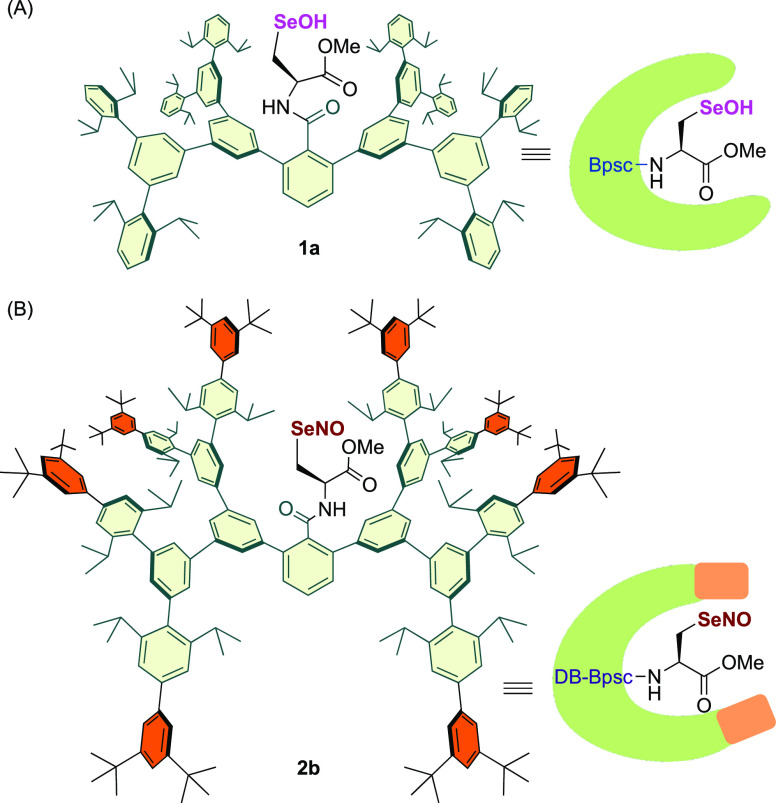
Cradled selenocysteines that bear the Bpsc group (A) and the DB-Bpsc group (B).

We thus synthesized Sec–SeH **4b** bearing the DB-Bpsc group^23^ as the starting material for Sec–SeNO **2b** by reduction of selenocystine derivative **3**(24) (Scheme 1). The structure of the DB-Bpsc molecular cradle was unequivocally determined by single-crystal X-ray diffraction analysis of the corresponding selenocysteine selenenyl iodide (Sec–SeI; **5b**). Sec–SeI **5b** was obtained from the treatment of Sec–SeH **4b** with *N*-iodosuccinimide (NIS).^25^ The single-crystal X-ray diffraction analysis (Figure 3) showed that the size of the DB-Bpsc group of **5b** is approximately 2.1 nm × 3.0 nm, which is much larger than that of Bpsc (1.7 nm × 2.3 nm; Figure S15).^22b^ The increased steric bulk of the peripheral moiety of the DB-Bpsc cradle can be expected to more effectively suppress the bimolecular decomposition of selenocysteine-derived reactive species than the Bpsc cradle.

**Scheme 1:**
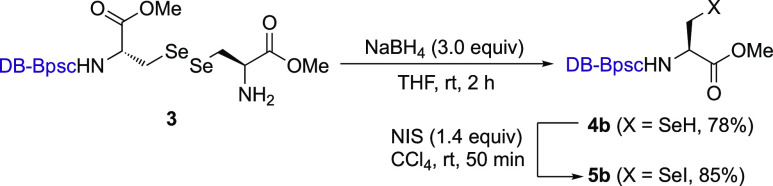
Synthesis of 4b and 5b

**Figure 3:**
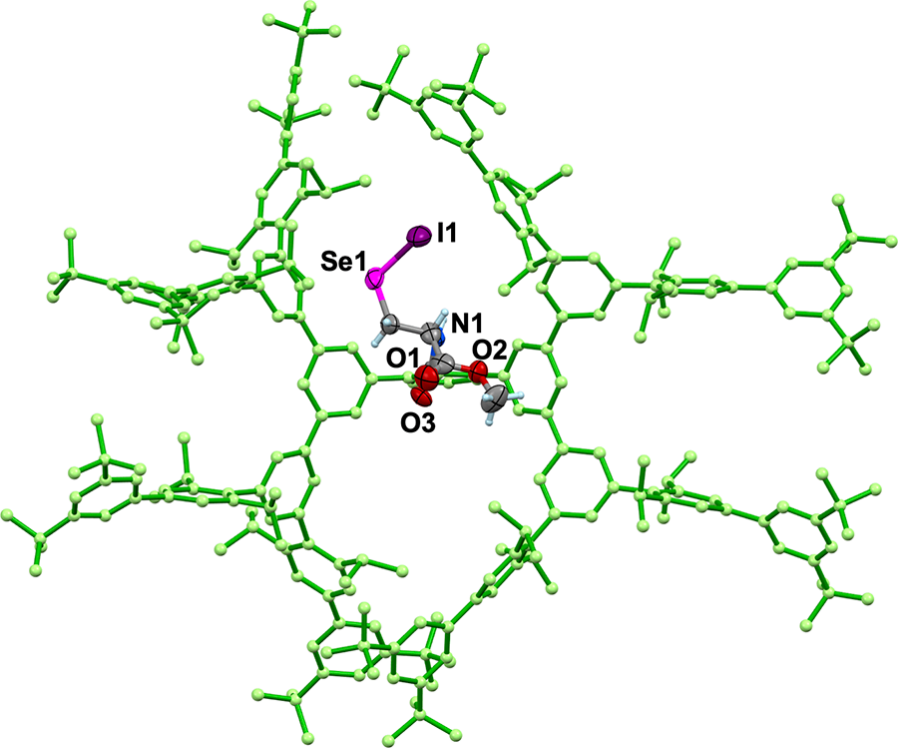
Crystal structure of **5b** (one of the two independent molecules).

The synthesis of Sec–SeNO **2b** by *Se*-nitrosation of Sec–SeH **4b** with organic nitrites was then examined, and the use of an ethanol solution of *t*-BuONO (8.0 equiv) as a nitrosating agent in THF-*d*_8_ at room temperature was identified as the optimal conditions (Figure 4a).^26^ After 13 h, the mixture gradually turned red and its ^1^H NMR monitoring indicated the formation of Sec–SeNO **2b** in 98% yield (Figure 4b). In the ^77^Se NMR spectrum in CDCl_3_, **2b** showed a signal at 2223 ppm (Figure 4c), which is in good agreement with those of the aryl-substituted *Se*-nitrososelenols (Figure 1c) that we have previously reported (Table S3).^14,15^ To the best of our knowledge, this is the first experimental evidence for the formation of a selenocysteine-derived *Se*-nitrososelenol. In the ^1^H NMR spectrum (THF-*d*_8_), the methylene protons adjacent to the selenium atom of **2b** were observed at 4.13 and 4.19 ppm, respectively. The downfield shifts of these methylene protons of Sec–SeNO **2b** compared to those of Sec–SeH **4b** (Table S2) indicate the presence of strong magnetic deshielding effects of the N=O group in **2b**. The UV–vis spectrum of the reddish reaction mixture in CDCl_3_ containing Sec–SeNO **2b**, which was generated by a reaction protocol similar to that shown in Figure 4a, exhibited an absorption maximum (λ_max_) at 446 nm (ε 167)^27^ (Figure 4d), which is assignable to the *n*–π* transition^28^ and consistent with the reported value for MeSeNO (λ_max_ = 440 nm) in an argon matrix at 10 K.^17^

**Figure 4:**
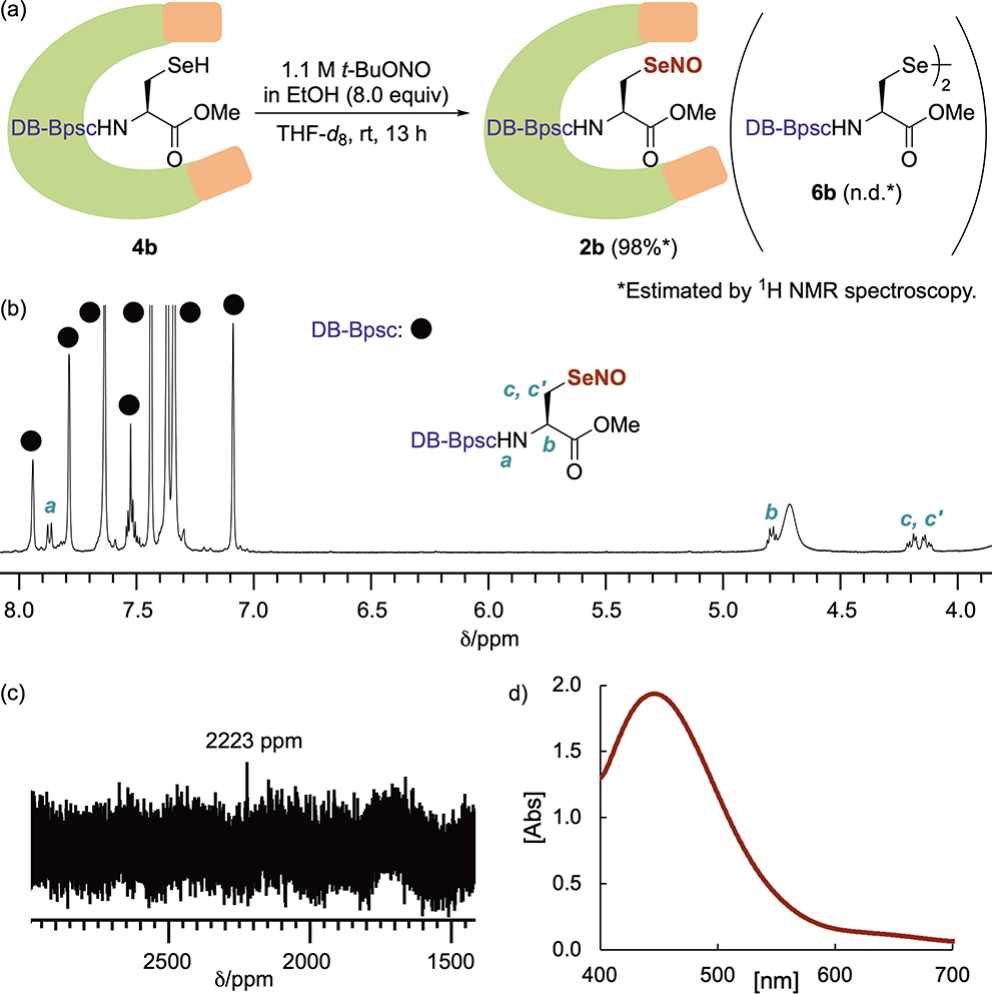
(a) *Se*-nitrosation of **4b**. (b) ^1^H NMR spectrum (500 MHz, THF-*d*_8_) of **2b**. (c) ^77^Se NMR spectrum (95 MHz, CDCl_3_) of **2b**. (d) UV–vis (CDCl_3_) spectrum of **2b**.

We attempted to isolate **2b** by the solvent removal-recrystallization sequence (Scheme 2a), but the resulting crystals contained not only **2b**, but also diselenide **6b** (**2b**:**6b** = 79:19).^29^ However, under conditions other than concentrated solutions, Sec–SeNO **2b** showed persistent stability; at a concentration of 8 mM in C_6_D_6_, no conversion from **2b** to **6b** was observed after 24 h at room temperature (Scheme 2b). Furthermore, **2b** showed essentially no decomposition in the presence of excess D_2_O (Scheme S14). Although *Se*-nitrososelenols have been proposed to be thermally labile due to facile Se–NO homolysis,^10,15,16^ these results indicate persistent stability for Sec–SeNOs, at least at physiological temperatures. This thermal stability of Sec–SeNO **2b** stands in sharp contrast to the behavior of Sec–SeOH **1a**, which undergoes spontaneous deselenation to the corresponding dehydroalanine^30,31^ at room temperature.^20a^ Due to the thermal instability of Sec–SeOHs, it has been proposed that the catalytic cycle of GPx must include a protective bypass mechanism that involves the intramolecular cyclization of the Sec–SeOH intermediates^32^ to the corresponding cyclic selenenyl amides^33^ to prevent inactivation by the deselenation from Sec–SeOHs. The stability of **2b** suggests that such a protective mechanism to prevent thermal degradation is not necessary for Sec–SeNOs generated in proteins at physiological temperatures.

**Scheme 2:**
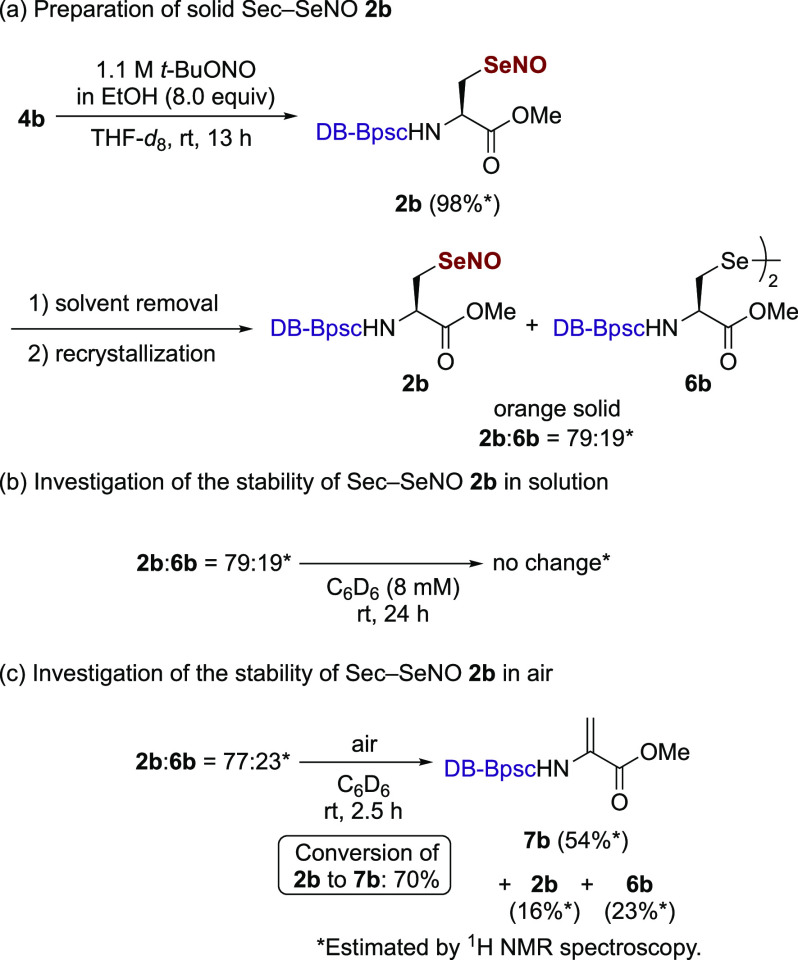
Investigation of the Stability of Sec–SeNO 2b

In contrast to the stability of Sec–SeNO **2b** under an inert gas atmosphere, **2b** was found to be sensitive to air. When a C_6_D_6_ solution of Sec–SeNO **2b** (77% purity)^34^ was exposed to air for 2.5 h, 70% of **2b** was converted to the corresponding dehydroalanine **7b** (Scheme 2c). This reaction is reminiscent of the thermal deselenation of Sec–SeOHs to dehydroalanines. It is likely that dehydroalanine **7b** was formed by initial oxidation of the selenium atom of **2b** and subsequent β elimination. These results indicate that Sec–SeNOs are more susceptible to oxidation than their sulfur analogues, Cys–SNOs, which are relatively stable in air.^12,35^ Under aerobic conditions, the SeNO modification of selenoproteins may lead to their degradation through oxidative deselenation.

Taniguchi et al. have shown that *S*-nitroso-*N*-acetyl-DL-penicillamine (SNAP)^35a^ induces the inactivation of GPx, probably through SeNO modification of the Sec residue, and that the inhibitory effect of SNAP on GPx is reversed by dithiothreitol (DTT).^6b,6c^ It has also been reported that SNAP-mediated inactivation of GPx finally forms a selenenyl sulfide bridge between Sec45 and Cys91. Benhar et al. have reported that exposure of TrxR to *S*-nitrosocysteine leads to an increased formation of a selenenyl sulfide bridge between Sec498 and Cys497.^8a^ These results can be feasibly interpreted by assuming that a reaction occurs between the SeNO group and the SH group of DTT or the Cys residue (Scheme 3a). We have already reported the similar reactivity of the nonselenocysteinyl *Se*-nitrososelenol Bpq-SeNO (Figure 1c) toward DTT and a thiol.^14^ Therefore, we subsequently carried out model studies of the reaction processes of the SeNO-modified selenoenzymes with cysteine thiols or DTT using the stable selenocysteine-derived compound **2b**. The ^1^H NMR monitoring of the reaction of Sec–SeNO **2b** (74% purity)^34^ with cysteine thiol **8** (3.0 equiv) in C_6_D_6_ at room temperature indicated the gradual conversion of **2b** to Sec–SeS–Cys **9** in 97% conversion yield after 14 h (Scheme 3b). These results support the notion that the formation of the selenenyl sulfide in the interactions of GPx or TrxR with RSNOs involves the preceding SeNO modification of the catalytic Sec residue. When **2b** (77% purity)^34^ was treated with DTT (3.0 equiv) in the presence of (*i*-Pr)_2_NEt (6.0 equiv) in C_6_D_6_ at room temperature for 20 min, **2b** was reduced to Sec–SeH **4b** in quantitative conversion yield (Scheme 3c), suggesting that the SeNO modification of selenoenzymes can be easily reversed by reducing thiols.

**Scheme 3:**
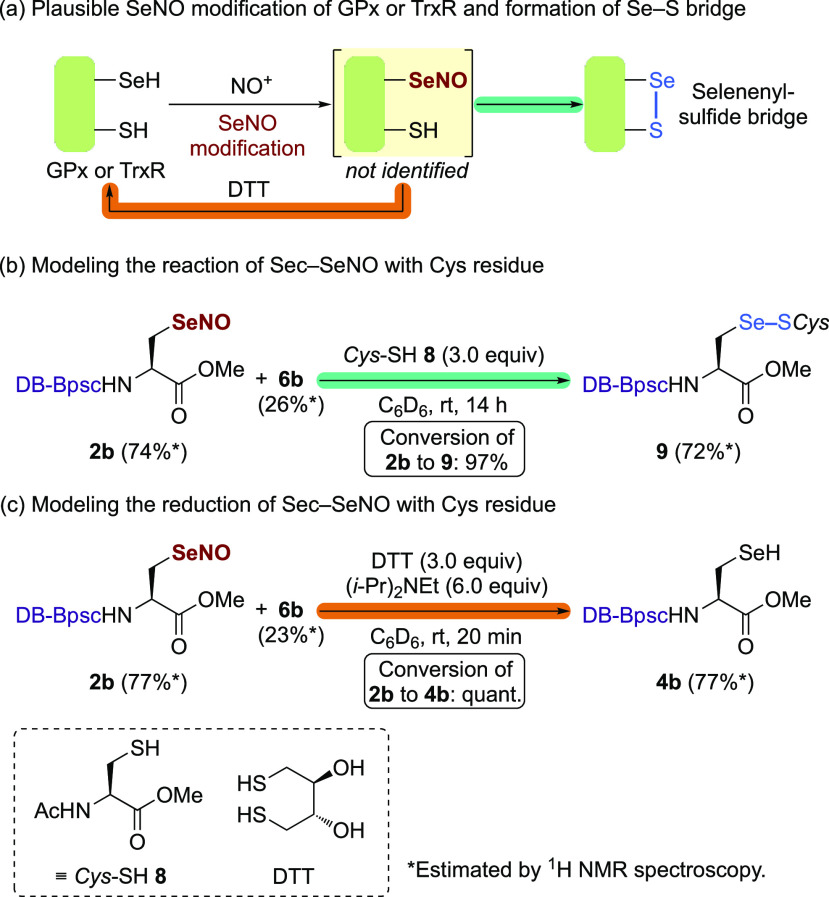
Model Reactions of Sec–SeNO with Thiols

We have previously reported that a *Se*-nitrososelenol undergoes intermolecular Se–Se bond formation more readily than a selenenic acid (Figure 1d).^15,19^ The high propensity of a Sec–SeNO to undergo diselenide formation was demonstrated experimentally by the behavior of Bpsc-subsituted Sec–SeNO **2a**, which is less bulky than DB-Bpsc-subsituted Sec–SeNO **2b**. When Sec–SeH **4a**(20a) was treated with an ethanol solution of *t*-BuONO (8.0 equiv) in THF-*d*_8_ at room temperature, the ^1^H NMR spectrum of the resulting solution after 22 h indicated the formation of a new compound derived from **4a**, most likely Sec–SeNO **2a**, albeit in a low yield of 26% (Figure 5a). The new compound showed spectral properties similar to those of DB-Bpsc-subsituted Sec–SeNO **2b** in the ^1^H NMR and UV/vis spectra (Figures S3 and S4), which is consistent with the formation of Sec–SeNO **2a**. However, the main product of this reaction was diselenide **6a** (55% NMR yield), along with the unreacted **4a** (19%) (Figure 5b). The bimolecular reaction of Sec–SeNO **2a** to produce diselenide **6a** was likely so fast that it was not fully suppressed by the Bpsc cradle, which can effectively protect Sec–SeOHs from self-condensation. The diselenide formation of Sec–SeNOs should be accompanied by the generation of NO. Given that *S*-nitrosothiols do not undergo disulfide formation very rapidly, not even when they do not carry bulky substituents,^12,36^ these results suggest that the spontaneous generation of NO from *Se*-nitrososelenols is much faster than that from *S*-nitrosothiols. Recently, various diselenides^37,38^ have been used as efficient catalysts for the generation of NO from Cys–SNOs.^39,40^ However, many reports^6,38,39,41^ have briefly summarized the mechanism for the NO generation by chemical equations in which selenols produced by the reduction of diselenides react with Cys–SNOs, leading to the release of two equivalents of NO (Figure 5c, top). Considering the ease of diselenide formation from *Se*-nitrososelenols, the detailed elementary-reaction processes of the catalytic NO generation can be rationalized in terms of an initial transnitrosation from Cys–SNOs to selenols to produce *Se*-nitrososelenols,^42^ which then rapid form the diselenide with concomitant release of NO (Figure 5c, bottom).

**Figure 5:**
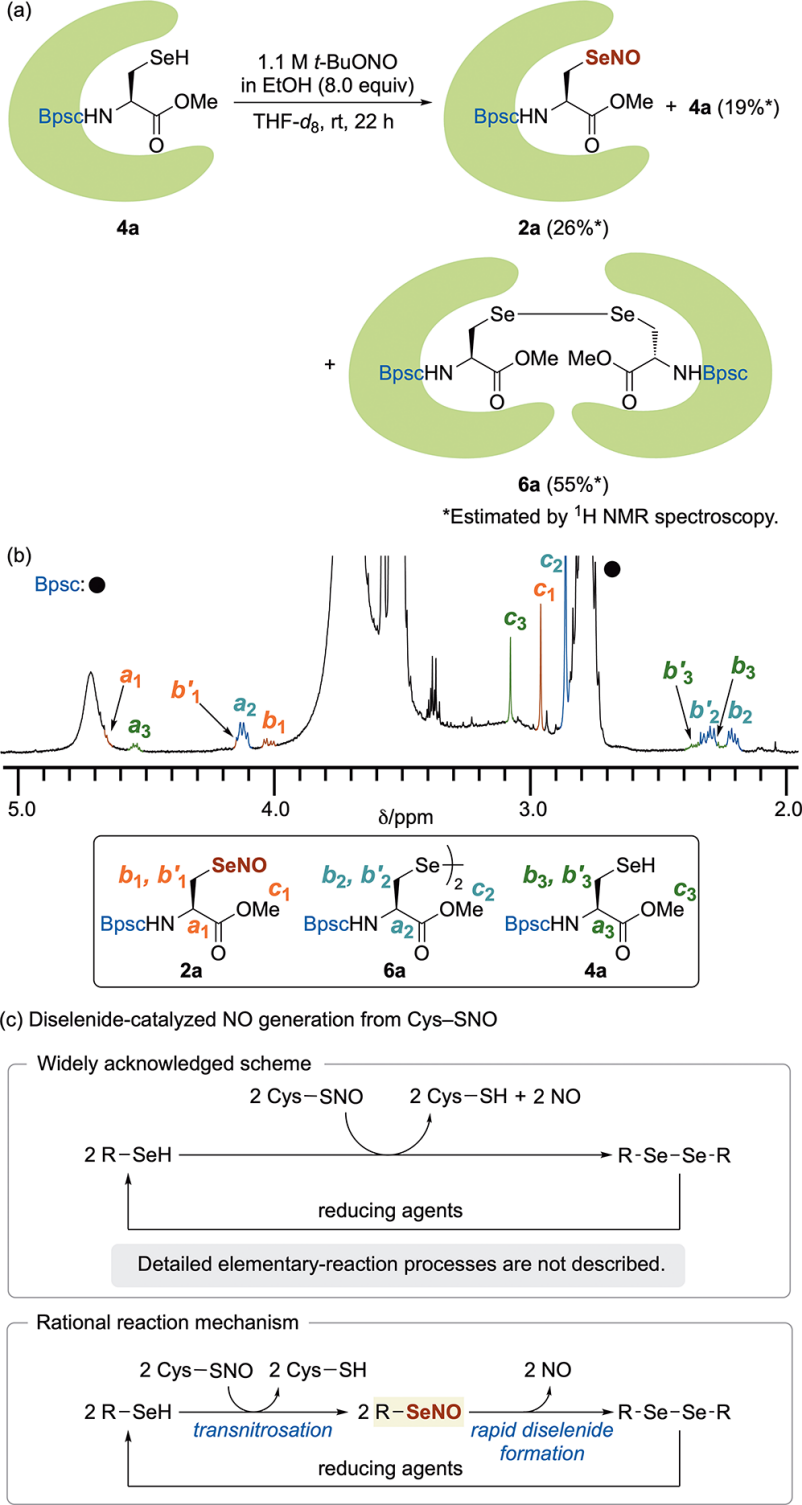
(a) *Se*-nitrosation of **4a**. (b) ^1^H NMR spectrum (500 MHz, THF-*d*_8_) of **2a**. (c) Diselenide-catalyzed NO generation from Cys–SNO.

In conclusion, we have presented the first synthesis of a *Se*-nitrososelenocysteine (Sec–SeNO) with persistent stability at room temperature by using a protective cradle with an expanded framework. The reaction processes involving this Sec–SeNO, along with those of its less bulky congener, provide important chemical information to elucidate the reaction mechanisms involving SeNO species in biological functions, as well as in the selenol-catalyzed NO generation from *S*-nitrosothiols.

## Supporting Information Available

Experimental procedures and spectral data (PDF)
