# Supplementary material for: Modeling Selenoprotein Se-Nitrosation: Synthesis of a Se-Nitrososelenocysteine with Persistent Stability
Source: J Am Chem Soc. 2023 Jun 2;145(26):14184–9. doi: 10.1021/jacs.3c03394 (PMC10326881; doi:10.1021/jacs.3c03394)
Supplement: Supplementary file 1 — ja3c03394_si_001.pdf [file ja3c03394_si_001.pdf]

Supporting Information for:

Modeling Selenoprotein *Se*-Nitrosation: Synthesis of a *Se*-Nitrososelenocysteine with Persistent Stability

Ryosuke Masuda, Satoru Kuwano, and Kei Goto*

Department of Chemistry, School of Science, Tokyo Institute of Technology, 2-12-1 Ookayama, Meguro-ku, Tokyo 152-8551, Japan

Contents

1. Experimental section **S2**

General experimental section S2

Synthesis of DB-Bpsc-OH (**S8**) S2

Synthesis of DB-Bpsc-Cl (**S9**) S3

Synthesis of selenocystine **3** S3

Synthesis of Sec–SeH **4b** S4

Synthesis of Sec–SeI **5b** S5

Se-Nitrosation of Sec–SeH **4a** bearing a Bpsc group S6

Optimization of generation of Sec–SeNO **2b** bearing a DB-Bpsc group S9

^{1}H NMR monitoring of the generation of Sec–SeNO **2b** bearing a DB-Bpsc group S11

Attempt to isolate Sec–SeNO **2b** S13

Measurement of the ^{77}Se NMR spectrum of Sec–SeNO **2b** S16

Measurement of the UV-vis spectrum of Sec–SeNO **2b** S17

Investigation of transnitrosation from GSNO to Sec–SeH **4b** S19

Investigation of the reactivity of Sec–SeNO **2b** S19

Investigation of the reactivity of Sec–SeNO **2b** toward D_2O S20

Reaction of Sec–SeNO **2b** with a cysteine thiol S21

Identification of dehydroalanine **7b** S22

Identification of selenenyl sulfide **9** S23

2. X-ray crystallographic analysis **S23**

3. NMR spectra **S27**

4. References **S34**

1. Experimental section

General experimental section

MBraun UNIlab glove box was used under an argon atmosphere. THF and Et_2O (anhydrous) were purchased from Kanto Chemical and passed through a Kayama Oxygen solvent purification system prior to use. CH_2Cl_2 (anhydrous) was purchased from Kanto Chemical and used as received. 1,4-Dioxane and CCl_4 were purchased from commercial sources and dried over 4ÅMS. EtOH (anhydrous) was purchased from Kanto Chemical and further dried over 4ÅMS. $CDCl_3$ was passed through a small column of neutral alumina prior to use. Et_3N and (i-Pr)$_2$NEt were purchased from commercial sources and distilled over KOH and then CaH_2. In the NMR experiments, THF-d_8 and $CDCl_3$ were purchased from commercial sources, distilled over CaH_2, and degassed through freeze-pump-thaw cycles. t-BuONO and EtONO were purchased from Sigma-Aldrich and TCI Chemical, respectively, which were transferred to J-Young tubes under argon atmosphere and degassed through freeze-pump-thaw cycles. Other chemicals were purchased from commercial sources and used as received. Silica gel column chromatography was performed using Kanto silica gel N60 or N40-50. ^{1}H NMR spectra were recorded on a JEOL ECX-500 and a JEOL ECZ-500, and the chemical shifts of ^{1}H are referenced to the residual proton signal of $CDCl_3$ (δ 7.26), THF-d_8 (δ 1.72), and C_6D_6 (δ 7.16). ^{13}C{^{1}H} NMR spectra were recorded on a JEOL ECX-500 or a JEOL ECZ-500, and the chemical shifts are given relative to $CDCl_3$ (δ 77.00) and C_6D_6 (δ 128.06). ^{77}Se{^{1}H} NMR spectra were recorded on a JEOL ECX-500, and the chemical shifts of ^{77}Se are referenced to Ph_2Se_2 (δ 480) as an external standard. All spectra were assigned with the aid of DEPT, COSY, HMQC, and HMBC NMR experiments. IR spectra were recorded on a JASCO FT/IR-4100. UV-vis spectra were recorded on a JASCO V-650 UV-vis spectrometer. High-resolution FD-TOF mass spectra were measured on a JEOL JMS-T100GCv "AccuTOF GCv". High-resolution ESI-TOF mass spectra were measured on a Bruker "micrOTOF II" at Open Facility Center in Tokyo Institute of Technology. Low-resolution MALDI-TOF mass spectrum was measured on a SHIMAZU AXIMA at Open Facility Center in Tokyo Institute of Technology. Melting points (m.p.) were measured with a Yanaco MP-S3 (uncorrected).

Synthesis of DB-Bpsc-OH (S8)

DB-Bpsc-OH (**S8**) was prepared by the reported procedure[1] from bromide **S1**,[2] as shown in Scheme S1.

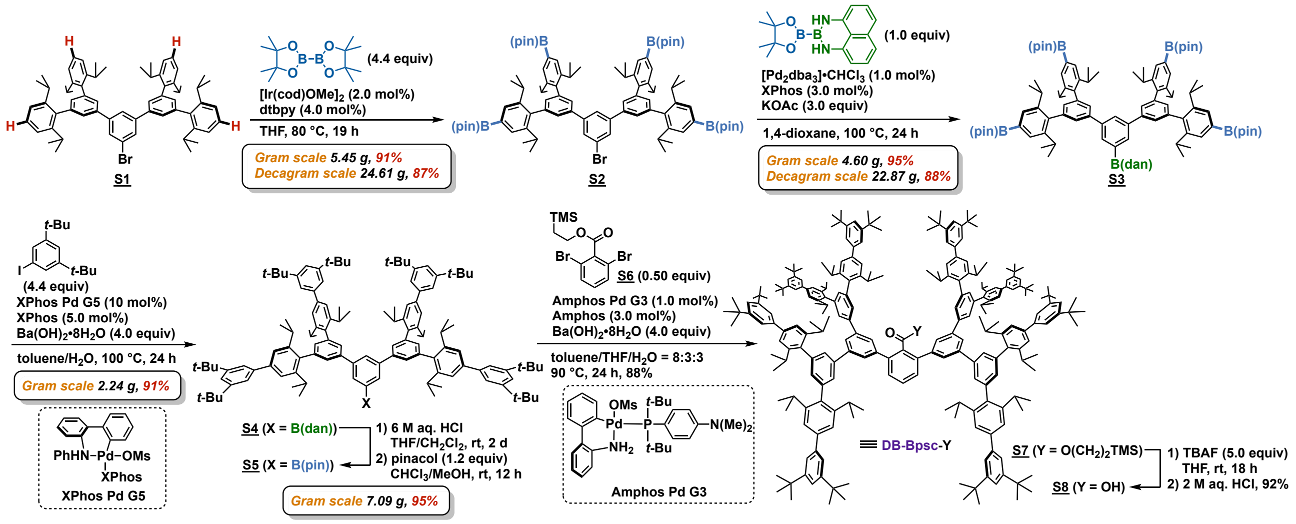

Scheme S1. Synthesis of DB-Bpsc-OH (**S8**).

Synthesis of DB-Bpsc-Cl (S9)

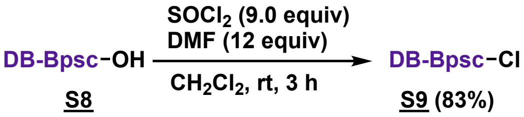

Scheme S2. Synthesis of DB-Bpsc-Cl (**S9**).

DB-Bpsc-OH (**S8**; 1.716 g, 0.5097 mmol) was placed in a 25 mL two-necked flask. After evacuated and backfilled with argon, CH_2Cl_2 (12.0 mL) and DMF (0.47 mL, 6.1 mmol) were added and then $SOCl_2$ (0.34 mL, 4.6 mmol) was slowly added. The reaction mixture was stirred at room temperature. After 3 h, the resulting mixture was concentrated in vacuo. The residue was dissolved in CH_2Cl_2 and concentrated in vacuo three times. The obtained solid was washed with CH_3CN and dried under vacuum at 70 °C to give DB-Bpsc-Cl (**S9**) as colorless crystals. Yield 1.425 g (0.4207 mmol, 83%).

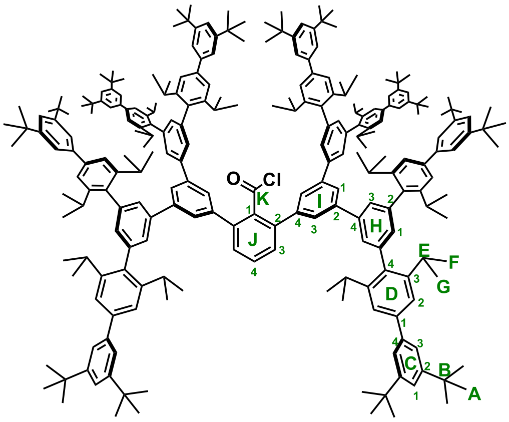

S9: colorless crystals; m.p. 254.0-255.0 °C (decomp.). **^{1}H NMR** (500 MHz, $CDCl_3$): δ 1.11 (d, J = 6.9 Hz, 48H, **F** or **G**), 1.18 (d, J = 6.9 Hz, 48H, **F** or **G**), 1.39 (s, 144H, **A**), 2.85 (septet, J = 6.8 Hz, 16H, **E**), 7.12 (br, 4H, **H1**), 7.35 (s, 16H, **D2**), 7.40 (d, J = 1.7 Hz, 16H, **C3**), 7.44 (t, J = 1.7 Hz, 8H, **C1**), 7.57 (d, J = 1.2 Hz, 8H, **H3**), 7.58-7.59 (m, 2H, **J3**), 7.63-7.66 (m, 1H, **J4**), 7.78 (d, J = 1.4 Hz, 4H, **I3**), 7.99 (br, 2H, **I1**); **^{13}C NMR** (125 MHz, $CDCl_3$): δ 24.2, 24.4 (q, **F** and **G**), 30.5 (d, **E**), 31.6 (q, **A**), 34.9 (s, **B**), 121.2 (d, **C1**), 121.89 (d, **D2**), 121.98 (d, **C3**), 126.1 (d, **I1**), 126.6 (d, **H3**), 126.9 (d, **I3**), 129.8 (d, **J3**), 130.7 (d, **H1**), 130.9 (d, **J4**), 137.1 (s, **J1**), 137.8 (s, **D4**), 139.1 (s, **J2**), 140.0 (s, **I2**), 140.2 (s, **D1**), 141.1 (s, **H2**), 141.8 (s, **I4**), 142.0 (s, **H4**), 142.3 (s, **C4**), 146.9 (s, **D3**), 150.8 (s, **C2**), 170.7 (s, **K**); **IR** (KBr): 3039, 2961, 2904, 1792, 1594, 1476, 1463, 1362, 1247, 869, 758, 713 cm^{-1}; **LRMS** (MALDI-TOF) *m/z* 3382.445 $[M]^+$ (calcd for $C_{251}H_{317}ClO$, 3382.444).

Synthesis of selenocystine 3

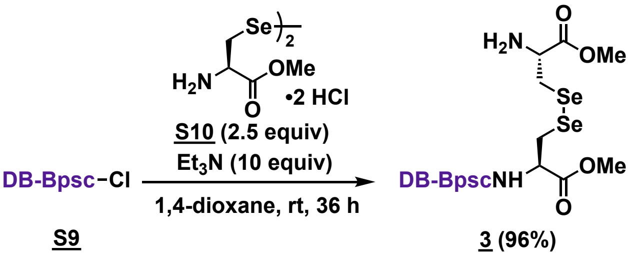

Scheme S3. Synthesis of selenocystine **3**.

Freshly prepared and powdered L-selenocystine methyl ester dihydrochloride (**S10**; 220 mg, 0.522 mmol), which

was prepared by the reported procedure,[3] was placed in a 25 mL Schlenk tube. After evacuated for 30 min and backfilled with argon, 1,4-dioxane (11.0 mL), DB-Bpsc-Cl (**S9**; 707 mg, 0.209 mmol) and then Et_3N (0.37 mL, 2.1 mmol) were added. The resulting reaction mixture was stirred at room temperature for 12 h before water was added. The two layers were separated and the aqueous layer was extracted with $CHCl_3$ (3×8 mL). The combined organic layer was washed with brine, dried over Na_2SO_4, and filtered. The filtrate was concentrated in vacuo and the crude product was purified by flash column chromatography on silica gel (hexane/CH_2Cl_2 = 4:1 then 1:1) to give **3** as yellow crystals. Yield 743 mg (0.200 mmol, 96%).

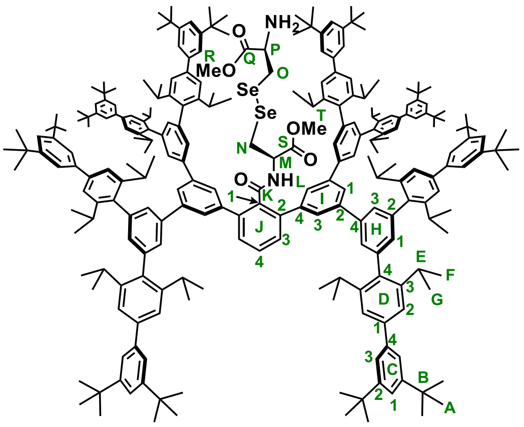

3: yellow crystals; m.p. 234.0-237.0 °C. **^{1}H NMR** (500 MHz, $CDCl_3$): δ 1.10-1.13 (m, 48H, **F** and **G**), 1.19-1.23 (m, 48H, **F** and **G**), 1.40 (s, 144H, **A**), 2.54 (dd, J = 13.5, 5.0 Hz, 1H, **N$_A$**), 2.86 (septet, J = 6.9 Hz, 16H, **E**), 2.90-2.93 (m, 2H, **O**), 3.06 (s, 3H, **T**), 3.06-3.10 (m, 1H, **N$_B$**), 3.37 (br t, J = 6.0 Hz, 1H, **P**), 3.43 (s, 3H, **R**), 4.82 (dt, J = 8.0, 4.3 Hz, 1H, **M**), 6.88 (d, J = 8.3 Hz, 1H, **L**), 7.10 (br, 4H, **H1**), 7.346-7.352 (m, 16H, **D2**), 7.41 (d, J = 2.0 Hz 16H, **C3**), 7.45 (t, J = 1.4 Hz, 8H, **C1**), 7.48-7.49 (m, 2H, **J3**), 7.52-7.55 (m, 1H, **J4**), 7.54 (br, 8H, **H3**), 7.76 (br, 4H, **I3**), 7.85 (br, 2H, **I1**); **^{13}C NMR** (125 MHz, $CDCl_3$): δ 24.2, 24.3, 24.5 (q, **F** and **G**), 29.9 (t, **N**), 30.5 (d, **E**), 31.6 (q, **A**), 35.0 (s, **B**), 35.7 (t, **O**), 51.0 (d, **M**), 51.7 (q, **T**), 51.9 (q, **R**), 54.3 (d, **P**), 121.2 (d, **C1**), 121.9 (d, **D2**), 122.0 (d, **C3**), 126.2 (d, **I1**), 126.9 (d, **H3**, overlapped, d, **I3**), 129.4 (d, **J4**), 130.3 (d, **J3**), 130.6 (d, **H1**), 134.4 (s, **J1**), 138.0 (s, **D4**), 140.3 (s, **J2**), 140.6 (s, **I2**), 141.0 (s, **D1**), 141.8 (s, **H2**, overlapped, s, **I4**), 141.9 (s, **H4**), 142.3 (s, **C4**), 147.0 (s, **D3**), 150.9 (s, **C2**), 168.2 (s, **K**), 169.9 (s, **S**), 173.8 (s, **Q**); **^{77}Se NMR** (95 MHz, $CDCl_3$): δ 284.8, 285.2; **IR** (KBr): 3415 (br), 3194, 3036, 2903, 2867, 1746, 1677, 1594, 1476, 1462, 1392, 1362, 1247, 869, 713 cm^{-1}; **HRMS** (FD-TOF) *m/z* 3710.4116 [M]$^+$ (calcd for $C_{259}H_{332}N_2O_5Se_2$, 3710.4116).

Synthesis of Sec–SeH 4b

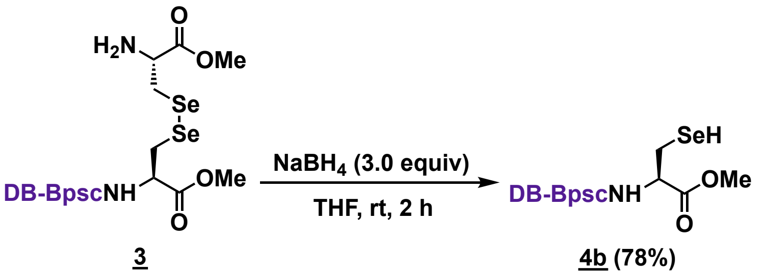

Scheme S4. Synthesis of Sec–SeH **4b**.

The following reaction and workup were performed under argon atmosphere. Unsymmetric selenocystine **3** (743 mg, 0.200 mmol) was placed in a 25 mL Schlenk tube. After evacuated and backfilled with argon, THF (10.0 mL) and $NaBH_4$ (22.7 mg, 0.600 mmol) were added. The resulting reaction mixture was stirred at room temperature for 2 h and it turned to a colorless suspension. After degassed 10% aq. NH_4Cl (2.0 mL) was added, the two layers were

separated and the aqueous layer was extracted with degassed $CHCl_3$ (3×8 mL) using Schlenk tubes. The combined organic layer was washed with degassed brine, dried over Na_2SO_4, and filtered through Celite on a glass filter. The filtrate was concentrated in vacuo and the obtained solid was washed with CH_3CN in a glove box and dried to give **4b** as colorless crystals. Yield 552 mg (0.156 mmol, 78%).

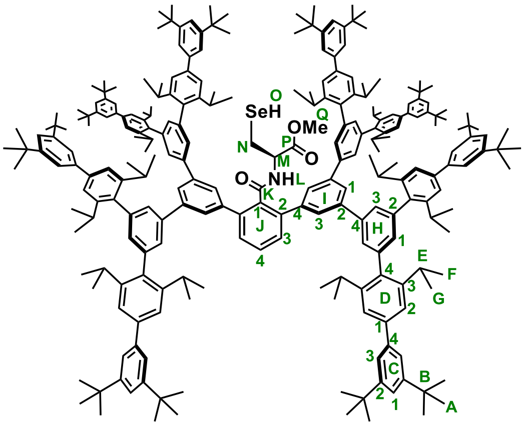

4b: colorless crystals; m.p. 257.0-259.0 °C. **^{1}H NMR** (500 MHz, $CDCl_3$): δ −1.41 (t, J = 8.0 Hz, 1H, **O**), 1.08-1.11 (m, 48H, **F** and **G**), 1.16-1.21 (m, 48H, **F** and **G**), 1.38 (s, 144H, **A**), 2.22-2.27 (m, 1H, **N$_A$**), 2.59-2.65 (m, 1H, **N$_B$**), 2.78-2.89 (m, 16H, **E**), 3.05 (s, 3H, **Q**), 4.76-4.79 (m, 1H, **M**), 6.53 (d, J = 7.7 Hz, 1H, **L**), 7.09 (br, 4H, **H1**), 7.328-7.334 (m, 16H, **D2**), 7.39 (d, J = 1.5 Hz, 16H, **C3**), 7.43 (t, J = 1.7 Hz, 8H, **C1**), 7.46-7.49 (m, 2H, **J3**), 7.50 (d, J = 1.4 Hz, 8H, **H3**), 7.51-7.55 (m, 1H, **J4**), 7.74 (d, J = 1.2 Hz, 4H, **I3**), 7.84 (br, 2H, **I1**); **^{13}C NMR** (125 MHz, $CDCl_3$): δ 19.2 (t, **N**), 24.17, 24.25, 24.41, 24.44 (q, **F** and **G**), 30.49, 30.55 (d, **E**), 31.57 (q, **A**), 35.0 (s, **B**), 52.2 (q, **Q**), 52.4 (d, **M**), 121.2 (d, **C3**), 121.9 (d, **D2**), 122.0 (d, **C1**), 126.3 (d, **I1**), 126.8 (d, **H3**, overlapped, d, **I3**), 129.4 (d, **J4**), 130.1 (d, **J3**), 130.6 (d, **H1**), 134.6 (s, **J1**), 137.9 (s, **D4**), 140.2 (s, **J2**), 140.5 (s, **I2**), 141.0 (s, **D1**), 141.7 (s, **I4**), 141.8 (s, **H2**), 142.0 (s, **H4**), 142.3 (s, **C4**), 146.96, 146.98 (s, **D3**), 150.9 (s, **C2**), 168.2 (s, **K**), 169.6 (s, **P**); **^{77}Se NMR** (95 MHz, $CDCl_3$): δ −82; **IR** (KBr): 3422 (br), 3037, 2962, 2905, 2868, 1752, 1680, 1594, 1477, 1462, 1362, 1247 869, 713 cm^{-1}; **HRMS** (FD-TOF) *m/z* 3529.4480 [M]$^+$ (calcd for $C_{255}H_{325}NO_3Se$, 3529.4474).

Synthesis of Sec–SeI 5b

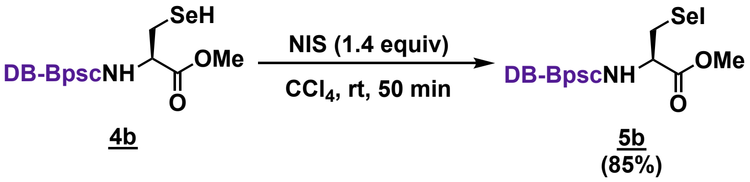

Scheme S5. Synthesis of Sec–SeI **5b**.

Sec–SeH **4b** (73.6 mg, 20.8 µmol) was placed in a 10 mL Schlenk tube. After evacuated and backfilled with argon, degassed CCl_4 (2.0 mL) and then freshly recrystallized NIS (6.6 mg, 29 µmol) were added. The resulting reaction mixture was stirred at room temperature for 50 min. The resulting purple mixture was filtered through Celite and then concentrated in vacuo. The obtained crude was triturated with CH_3CN to give **5b** as purple crystals. Yield 64.7 mg (17.7 µmol, 85%).

5b: purple crystals; m.p. 257.0-258.5 °C. **^{1}H NMR** (500 MHz, $CDCl_3$): δ 1.09-1.12 (m, 48H, **F** and **G**), 1.18-1.22 (m, 48H, **F** and **G**), 1.38 (s, 144H, **A**), 2.73 (dd, J = 14.3, 5.5 Hz, 1H, **N$_A$**), 2.78-2.89 (m, 16H, **E**), 3.05 (s, 3H, **P**), 3.12 (dd, J = 13.6, 3.5 Hz, 1H, **N$_B$**), 4.72-4.75 (m, 1H, **M**), 6.64 (d, J = 8.0 Hz, 1H, **L**), 7.10 (br, 4H, **H1**), 7.334-7.341 (m, 16H, **D2**), 7.39 (d, J = 2.0 Hz, 16H, **C3**), 7.44 (t, J = 1.5 Hz, 8H, **C1**), 7.47-7.49 (m, 2H, **J3**), 7.51 (d, J = 1.5 Hz, 8H, **H3**), 7.51-7.55 (m, 1H, **J4**), 7.73 (d, J = 1.0 Hz, 4H, **I3**), 7.85 (br, 2H, **I1**); **^{13}C NMR** (125 MHz, $CDCl_3$):

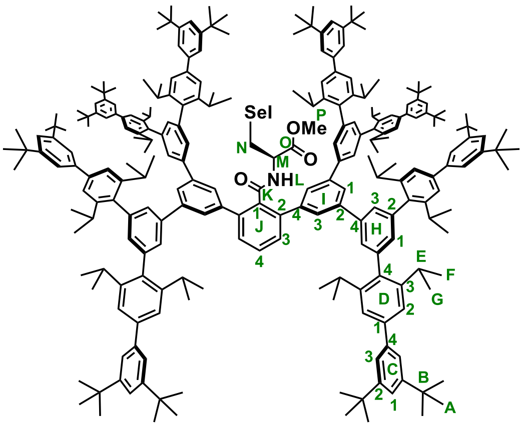

δ 24.2, 24.3, 24.41, 24.43 (q, **F** and **G**), 29.7 (t, **N**), 30.5, 30.6 (d, **E**), 31.6 (q, **A**), 35.0 (s, **B**), 51.4 (d, **M**), 52.0 (q, **P**), 121.2 (d, **C3**), 121.9 (d, **D2**), 122.0 (d, **C1**), 126.2 (d, **I1**), 126.77 (d, **H3**), 126.84 (d, **I3**), 129.5 (d, **J4**), 130.2 (d, **J3**), 130.6 (d, **H1**), 134.1 (s, **J1**), 137.9 (s, **D4**), 140.1 (s, **J2**), 140.4 (s, **I2**), 141.0 (s, **D1**), 141.6 (s, **I4**), 141.8 (s, **H2**), 141.9 (s, **H4**), 142.3 (s, **C4**), 147.0 (s, **D3**), 150.9 (s, **C2**), 168.2 (s, **K**), 168.9 (s, **O**); ^{77}**Se NMR** (95 MHz, $CDCl_3$): δ 385; **IR** (KBr): 3417, 3038, 2961, 2904, 2867, 1750, 1594, 1477, 1462, 1383, 1362, 1247, 869, 713 cm^{-1}; **UV-vis** ($CHCl_3$, 298 K) λ_{max} 492 nm (ε = 55); **HRMS** (ESI-TOF) *m/z* 3690.3098 [M+Cl]$^-$ (calcd for $C_{255}H_{324}ClINO_3Se$, [M+Cl]$^-$, 3590.3135).

***Se*-Nitrosation of Sec–SeH 4a bearing a Bpsc group**

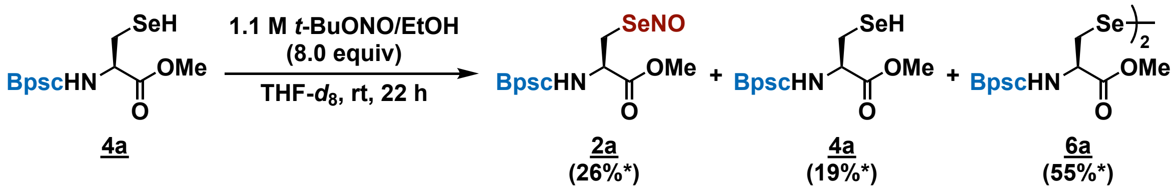

Scheme S6. *Se*-Nitrosation of Sec–SeH **4a** bearing a Bpsc group.

A stock solution of *t*-BuONO (90%, 0.60 mL, 4.5 mmol) in dried EtOH (4.0 mL) was prepared prior to the reaction. Sec–SeH **4a** (39.9 mg, 19.8 μmol), which was prepared by the reported procedure,[4] was placed in a 5 mm o/d NMR tube with a J-Young valve. After evacuated and backfilled with argon, freshly distilled and degassed THF-d_8 (0.60 mL) and the stock solution of *t*-BuONO (1.1 M, 79 μL, 90 μmol) were added. The resulting reaction mixture was degassed through freeze-pump-thaw cycles, and the tube was then flushed with argon. After 22 h, the color of the mixture turned to reddish orange. A ^{1}H NMR spectrum of the obtained mixture was recorded and the NMR yields of Sec–SeNO **2a**, Sec–SeH **4a**, and the corresponding diselenide **6a** were determined as 26%, 19%, and 55%, respectively (**Figure S1** and **S3**). The identification of several signals was conducted by the ^{1}H-^{1}H COSY spectrum (**Figure S2**). The signals of **4a** and **6a** in the ^{1}H NMR spectrum were in agreement with those of the isolated sample.[4] In a glove box (N_2), the mixture was transferred to a quartz cell and a UV-vis spectrum was recorded at 25 °C after 5 min (**Figure S4**). **UV-vis** (THF-d_8, 298 K) λ_{max} 434 nm (sh). Comparison of the ^{1}H NMR chemical shifts **2a** with those of **4a** is shown in Table S1.

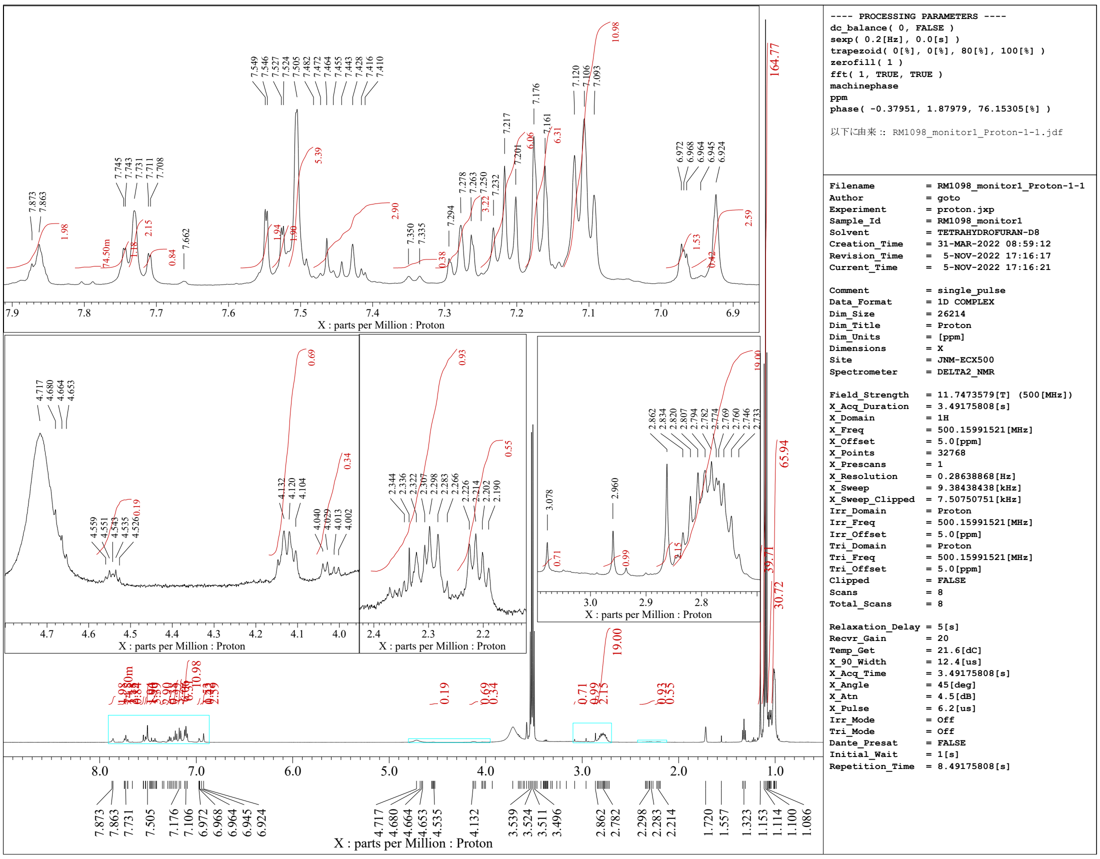

Figure S1. ^{1}H NMR (500 MHz, THF-d_8) spectrum of the mixture obtained in Scheme S6.

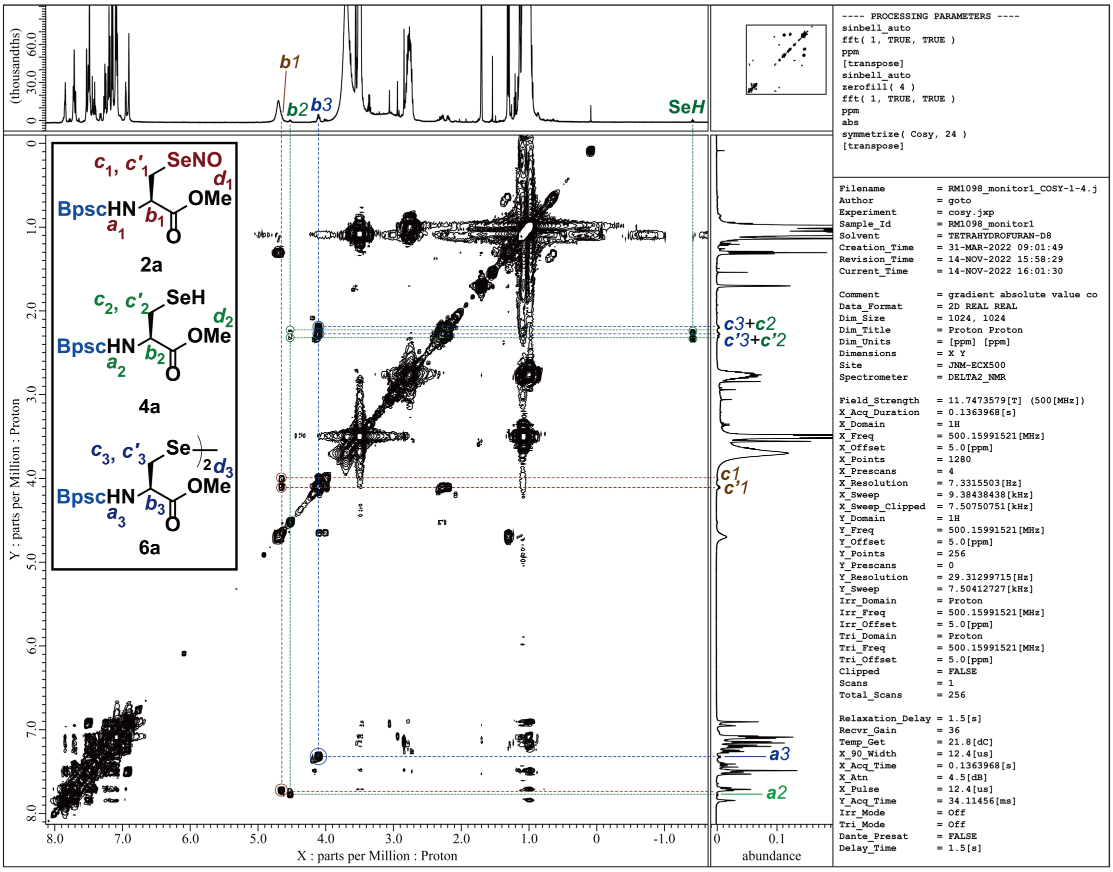

Figure S2. ^{1}H-^{1}H COSY (500 MHz, THF-d_8) spectrum of the mixture obtained in Scheme S6.

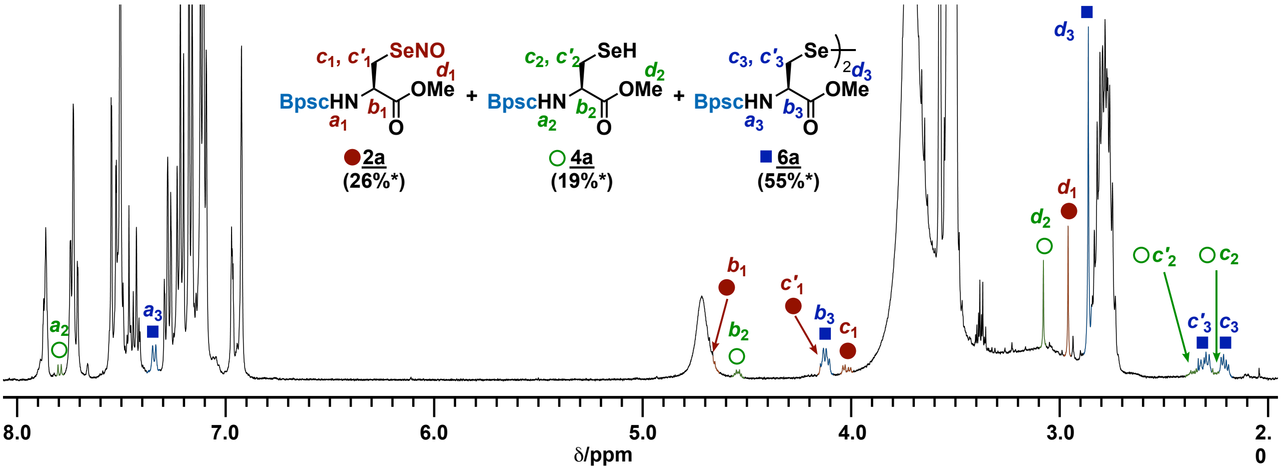

Figure S3. Assignment of the signal in ^{1}H NMR (500 MHz, THF-d_8) spectrum of the mixture obtained in Scheme S6.

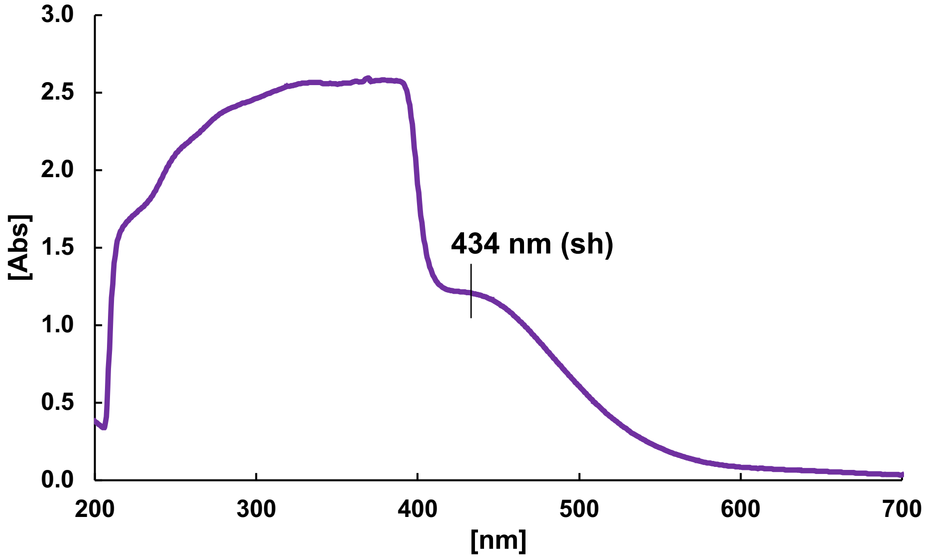

Figure S4. UV-vis (THF-d_8, 298 K) spectrum of the mixture obtained in Scheme S6.

Table S1. Comparison of the chemical shifts of **2a** and **4a** on ^{1}H NMR (500 MHz, THF-d_8).

	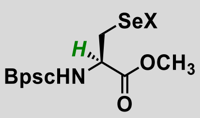 methine proton (ppm)	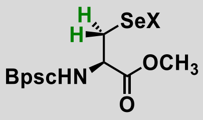 methylene protons (ppm)
**2a** (X = NO)	ca. 4.6 (m)	4.02 (dd, J = 13.5, 5.5 Hz)<br>4.10-4.13 (m)
**4a** (X = H)[4]	4.54 (dt, J = 8.0, 4.0 Hz)	2.26-2.31 (m)<br>2.33-2.38 (m)

Optimization of generation of Sec–SeNO 2b bearing a DB-Bpsc group

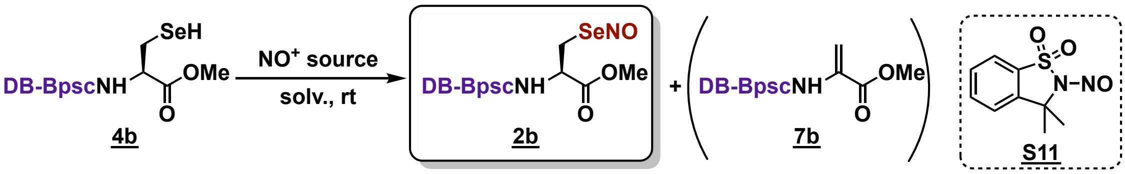

entry	NO+ source	NO+ (equiv)	solv.	time	conv. (%)	results
1	1.1 M *t*-BuONO/EtOH	8.0	$CDCl_3$	77 h	>98	2b (78%)* + 7b (18%)*
2	*t*-BuONO	8.0	$CDCl_3$	3 h	>99	2b (trace)* + 7b (95%)*
3	*t*-BuONO + TFA (2 equiv)	8.0	$CDCl_3$[a]	7 min	>99	7b (>99%)*
4	15% EtONO/EtOH	8.0	$CDCl_3$	4 h	>99	2b (35%)* + 7b (23%)*
5	15% EtONO/EtOH	1.0	$CDCl_3$	16 h	>99	2b (35%)* + 7b (24%)*
6	15% EtONO/EtOH	1.0	C_6D_6	72 h	0	No reaction
7	S11	1.2	$CDCl_3$	48 h	0	No reaction
8	S11 + HCl (1 equiv)	1.2	$CDCl_3$[a]	1 h	>99	Sec–SeCl was formed.
9	*t*-BuONO	8.0	THF-d_8	45 h	>99	2b (74%)* + 7b (25%)*
10	1.1 M *t*-BuONO/EtOH	8.0	THF-d_8	20 h	>99	2b (98%)*

[a] Stored with CaH_2. *Estimated by ^{1}H NMR spectroscopy.

Scheme S7. Optimization of generation of Sec–SeNO **2b** bearing a DB-Bpsc group.

General procedure: NO+ source **S11** was prepared by the reported procedure.[5] Sec–SeH **4b** was placed in a 5 mm o/d NMR tube with a J-Young valve. After evacuated and backfilled with argon, a freshly distilled and degassed solvent, bis(trimethylsilyl)methane (1 μL) as an internal standard, and an NO+ source were added. In entries 3 and 8 (Scheme S7), CF_3CO_3H or HCl in 1,4-dioxane was subsequently added, respectively. The resulting mixture was degassed through freeze-pump-thaw cycles, and the tube was then flushed with argon. The ^{1}H NMR spectra at selected times were recorded and the NMR yields of Sec–SeNO **2b** and/or the corresponding dehydroalanine **7b** were estimated. For example, copies of the ^{1}H NMR spectra recorded in entry 1 are shown in Figure S5.

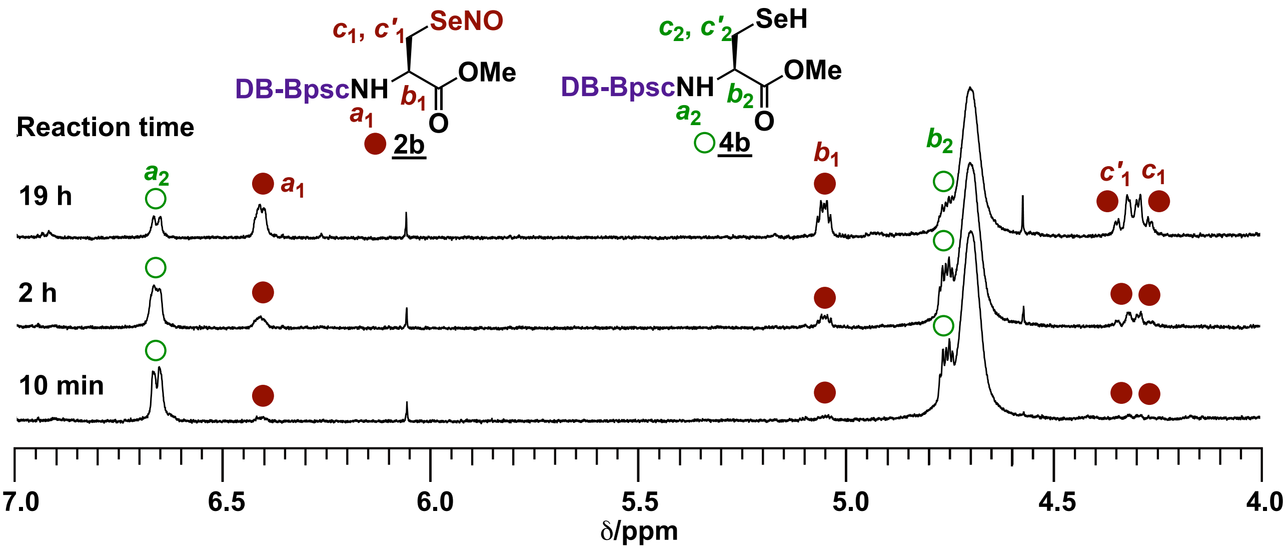

Figure S5. Monitoring of the mixture obtained in entry 1, Scheme S7, by ^{1}H NMR (500 MHz, $CDCl_3$) spectroscopy.

(a) entry 1

A stock solution of *t*-BuONO (90%, 300 μL, 2.27 mmol) in dried EtOH (2.0 mL) was prepared prior to the reaction.

Reagents: **4b** (12.0 mg, 3.39 μmol), *t*-BuONO in EtOH (1.1 M, 24 μL, 27 μmol), $CDCl_3$ (0.60 mL)

Result: The NMR yields of **2b** and **7b** were determined as 78% and 18%, respectively.

(b) entry 2

Reagents: **4b** (28.7 mg, 8.14 μmol), *t*-BuONO (90%, 8.6 μL, 65 μmol), $CDCl_3$ (0.80 mL)

Result: The NMR yields of **2b** and **7b** were determined as trace and 95%, respectively.

(c) entry 3

Reagents: **4b** (22.3 mg, 6.31 μmol), *t*-BuONO (90%, 6.0 μL, 51 μmol), CF_3CO_2H (0.8 μL, 10 μmol, 1.6 equiv), $CDCl_3$ (0.80 mL, stored with CaH_2)

Result: **7b** was formed in >99% NMR yield.

(d) entry 4

Reagents: **4b** (13.2 mg, 3.74 μmol), EtONO in EtOH (1.6 M, 19 μL, 30 μmol), $CDCl_3$ (0.80 mL)

Result: The NMR yields of **2b** and **7b** were determined as 35% and 23%, respectively.

(e) entry 5

Reagents: **4b** (15.2 mg, 4.30 μmol), EtONO in EtOH (1.6 M, 2.7 μL, 4.3 μmol), $CDCl_3$ (0.80 mL)

Result: The NMR yields of **2b** and **7b** were determined as 35% and 24%, respectively.

(f) entry 6

Reagents: **4b** (11.4 mg, 3.23 μmol), EtONO in EtOH (1.6 M, 2.0 μL, 3.2 μmol), C_6D_6 (0.60 mL)

Result: No reaction.

(g) entry 7

Reagents: **4b** (29.6 mg, 8.39 μmol), **S11** (2.3 mg, 10 μmol), $CDCl_3$ (0.60 mL)

Result: No reaction.

(h) entry 8

Reagents: **4b** (27.1 mg, 7.67 μmol), **S11** (2.1 mg, 9.2 μmol), HCl in 1,4-dioxane (4 M, 2 μL, 8 μmol), $CDCl_3$ (1.0 mL, stored with CaH_2)

Result: The corresponding Sec–SeCl was formed quantitatively.

(i) entry 9

A stock solution of *t*-BuONO (90%, 300 μL, 2.27 mmol) in dried EtOH (2.0 mL) was prepared prior to the reaction.

Reagents: **4b** (10.9 mg, 3.09 μmol), *t*-BuONO (90%, 22 μL, 25 μmol), $CDCl_3$ (0.70 mL)

Result: The NMR yields of **2b** and **7b** were determined as 74% and 25%, respectively.

(j) entry 10

The experimental detail was described in Scheme S8 (vide infra).

^{1}H NMR monitoring of the generation of Sec–SeNO 2b bearing a DB-Bpsc group

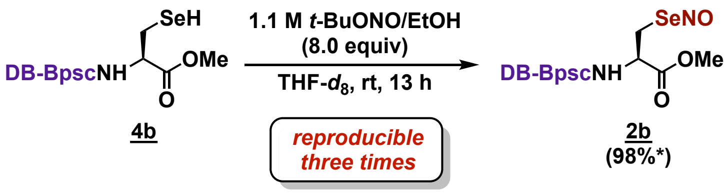

run	4b (mg)	NMR yield of 2b (%)
1	39.9	98
2	42.9	98
3	41.4	98

Scheme S8. ^{1}H NMR monitoring of the generation of Sec–SeNO **2b** bearing a DB-Bpsc group.

(a) run 1

A stock solution of *t*-BuONO (90%, 300 μL, 2.27 mmol) in dried EtOH (2.0 mL) was prepared prior to the reaction. Sec–SeH **4b** (39.9 mg, 11.3 μmol) was placed in a 5 mm o/d NMR tube with a J-Young valve. After evacuated and backfilled with argon, freshly distilled and degassed THF-d_8 (0.60 mL) and the stock solution of *t*-BuONO (1.1 M, 79 μL, 90 μmol) were added. The resulting mixture was degassed through freeze-pump-thaw cycles, and the tube was then flushed with argon. The ^{1}H NMR spectra at selected times were recorded. After 13 h, the formation of Sec–SeNO **2b** in 98% NMR yield was observed by ^{1}H NMR spectroscopy (**Figure S6** and **S7**). Comparison of the ^{1}H NMR chemical shifts **2b** with those of **4b** is shown in Table S2.

This experiment was reproduced three times.

(b) run 2

Reagents: **4b** (42.9 mg, 12.1 μmol), *t*-BuONO/EtOH (1.1 M, 85 μL, 97 μmol), $CDCl_3$ (0.60 mL)

Result: **2b** (98%)

(c) run 3

Reagents: **4b** (41.4 mg, 11.7 μmol), *t*-BuONO/EtOH (1.1 M, 82 μL, 94 μmol), $CDCl_3$ (0.60 mL)

Result: **2b** (98%)

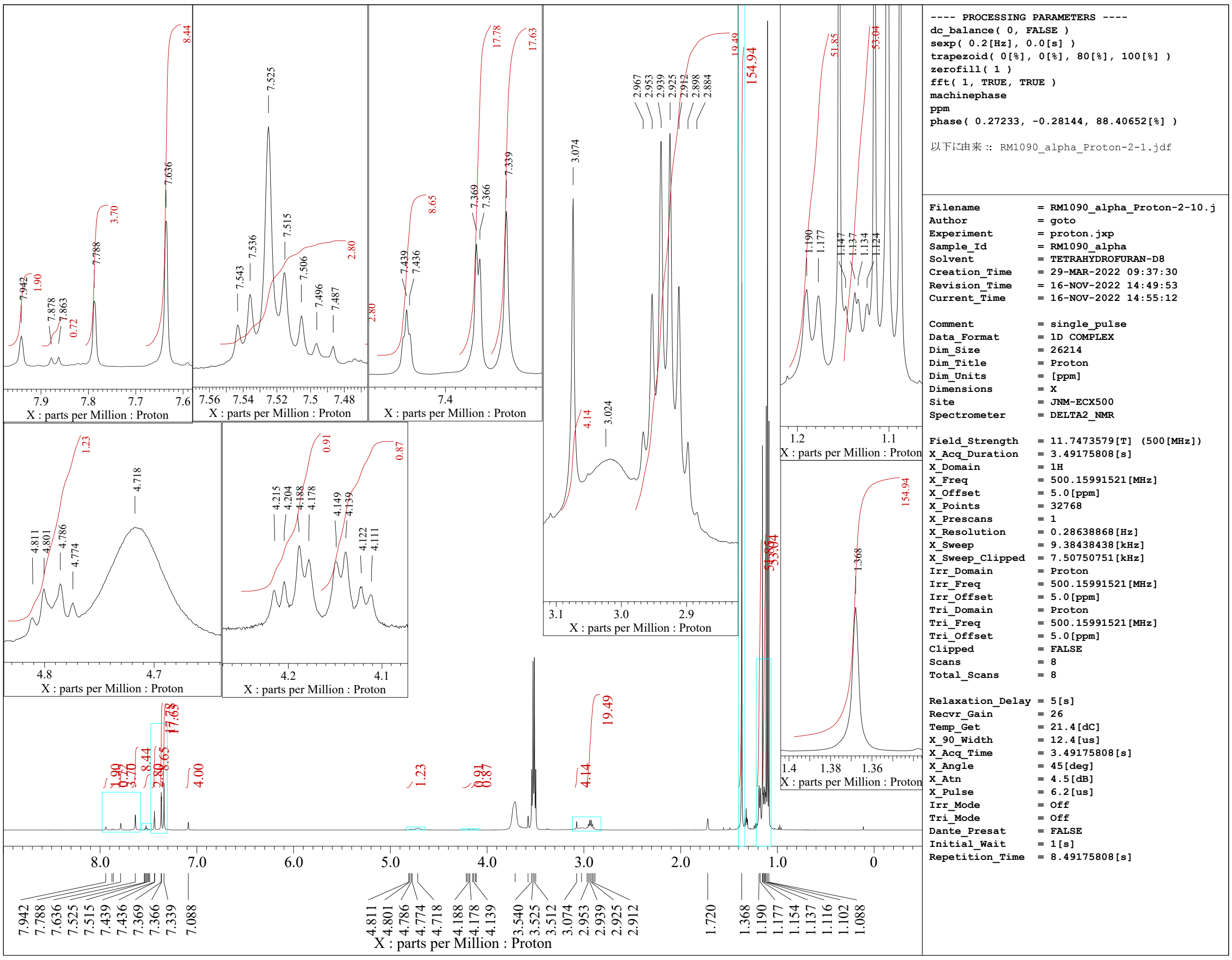

Figure S6. ^{1}H NMR (500 MHz, THF-d_8) spectrum of the mixture obtained in run 1, Scheme S8.

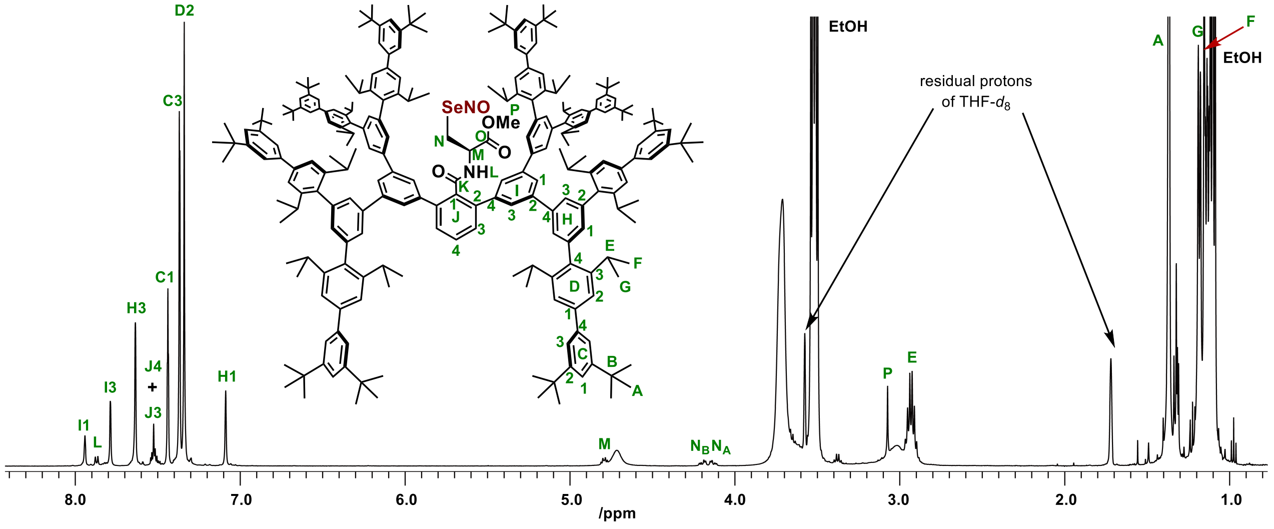

Figure S7. Assignment of the signal in ^{1}H NMR (500 MHz, THF-d_8) spectrum of the mixture obtained in run 1, Scheme S8.

Table S2. Comparison of the chemical shifts of **2b** and **4b** on ^{1}H NMR (500 MHz, THF-d_8).

	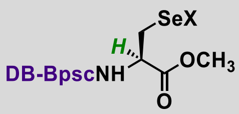 methine proton (ppm)	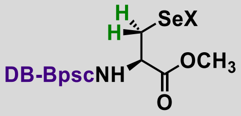 methylene protons (ppm)
**2b** (X = NO)	4.79 (dt, J = 7.5, 5.0 Hz)	4.13 (dd, J = 13.8, 5.0 Hz)<br>4.19 (dd, J = 13.3, 5.5 Hz)
**4b** (X = H)	4.62 (dt, J = 7.0, 6.0 Hz)	2.30-2.37 (m)<br>2.44-2.50 (m)

Attempt to isolate Sec–SeNO 2b

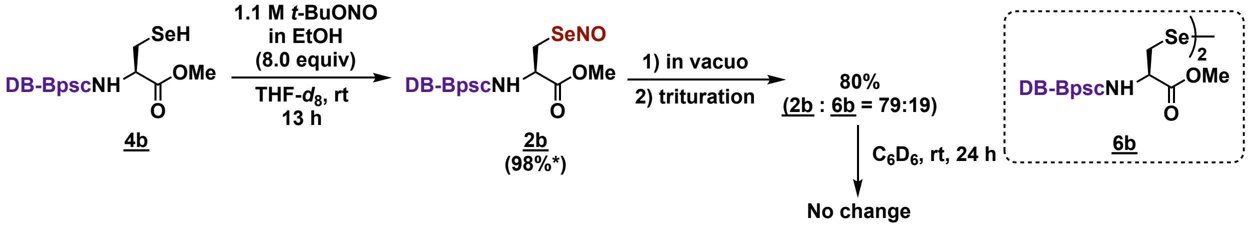

Scheme S9. Attempt to isolate Sec–SeNO **2b**.

The mixtures which were obtained in runs 1–3, Scheme S8, were used for this experiment. The following operations were performed in a glove box (Ar). The obtained mixtures in J-Young NMR tubes were combined and transferred to a 20 mL round bottom flask by using benzene. After approximately 90% of the solvent was removed, the resulting brownish orange mixture was triturated with CH_3CN to give the mixture of **2b** with **6b** as orange solids. Yield 99.8 mg (28 µmol, 80%, if pure **2b**). By ^{1}H NMR (C_6D_6) spectroscopy, the content of Sec–SeNO **2b** and the corresponding diselenide **6b** was estimated to be 79% and 19%, respectively (**Figure S8** and **Figure S9**), which did not change after 24 h. The ^{13}C NMR spectrum of **2b** was recorded by using this mixture (**Figure S10**).

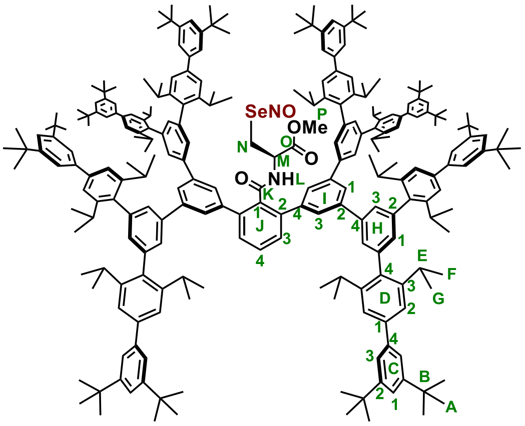

2b: **^{1}H NMR** (500 MHz, THF-d_8): δ 1.12-1.15 (m, 48H, **F** and **G**), 1.18-1.19 (m, 48H, **F** and **G**), 1.37 (s, 144H, **A**), 2.88-2.97 (m, 16H, **E**), 3.07 (s, 3H, **P**), 4.13 (dd, J = 13.8, 5.0 H, $\mathbf{N_A}$), 4.19 (dd, J = 13.3, 5.5 Hz, $\mathbf{N_B}$), 4.79 (dt, J = 7.5, 5.0 Hz, **M**), 7.09 (br, 4H, **H1**), 7.34 (s, 16H, **D2**), 7.37 (d, J = 1.5 Hz, 16H, **C3**), 7.44 (br t, J = 1.5 Hz, 8H, **C1**), 7.49-7.54 (m, 3H, **J3** and **J4**), 7.64 (br, 8H, **H3**), 7.79 (br, 4H, **I3**), 7.87 (d, J = 7.5, 1H, **L**), 7.94 (br, 2H, **I1**); **^{13}C NMR** (125 MHz, C_6D_6): δ 24.50, 24.53, 24.8 (q, **F** and **G**), 31.2 (d, **E**), 31.8 (q, **A**), 33.1 (t, **N**), 35.1 (s, **B**), 51.7 (q, **P**), 53.4 (d, **M**), 121.5 (d, **C1**), 122.6 (d, **C3**), 122.7 (d, **D2**), 126.4 (d, **I1**), 127.6 (d, **H3**), 128.5 (d, **I3**), 129.4 (d, **J4**), 129.6 (d, **J3**), 131.3 (d, **H1**), 136.2 (s, **J1**), 138.6 (s, **D4**), 140.1 (s, **J2**), 141.89 (s, **I2**), 141.93 (s, **I4**), 142.0 (s, **H2**), 142.5 (s, **H4**), 143.0 (s, **D1**), 143.6 (s, **C4**), 147.6 (s, **D3**), 151.4 (s, **C2**), 167.9 (s, **K**), 169.4 (s, **O**); **IR** (KBr): 2960, 2867, 1747, 1681, 1594, 1462, 1392, 1362, 1311, 1247 cm^{-1}.

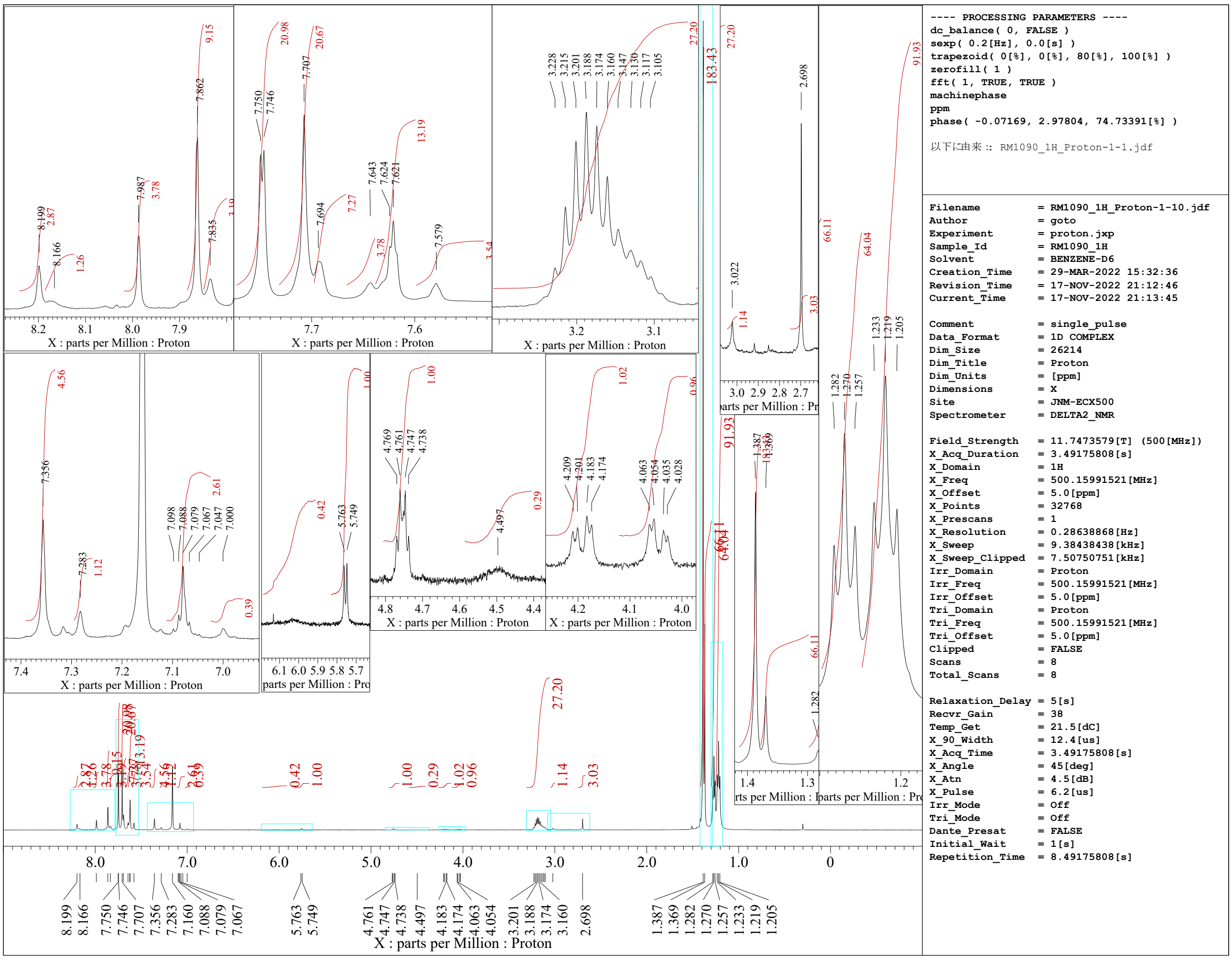

Figure S8. ^{1}H NMR (500 MHz, C_6D_6) spectrum of the mixture obtained in Scheme S9.

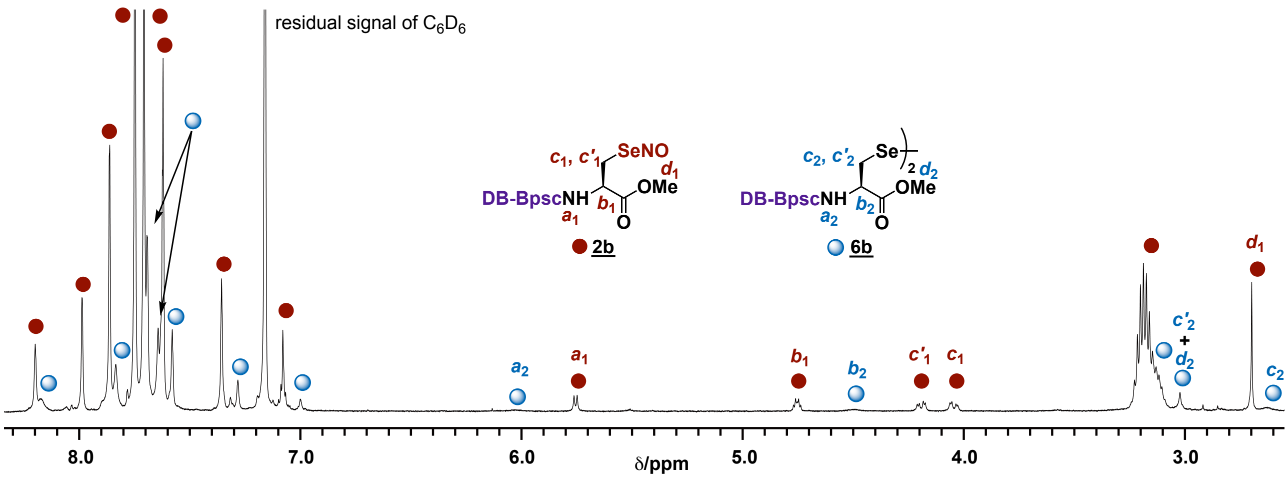

Figure S9. Assignment of the signals in ^{1}H NMR (500 MHz, C_6D_6) spectrum of the mixture obtained in Scheme S9.

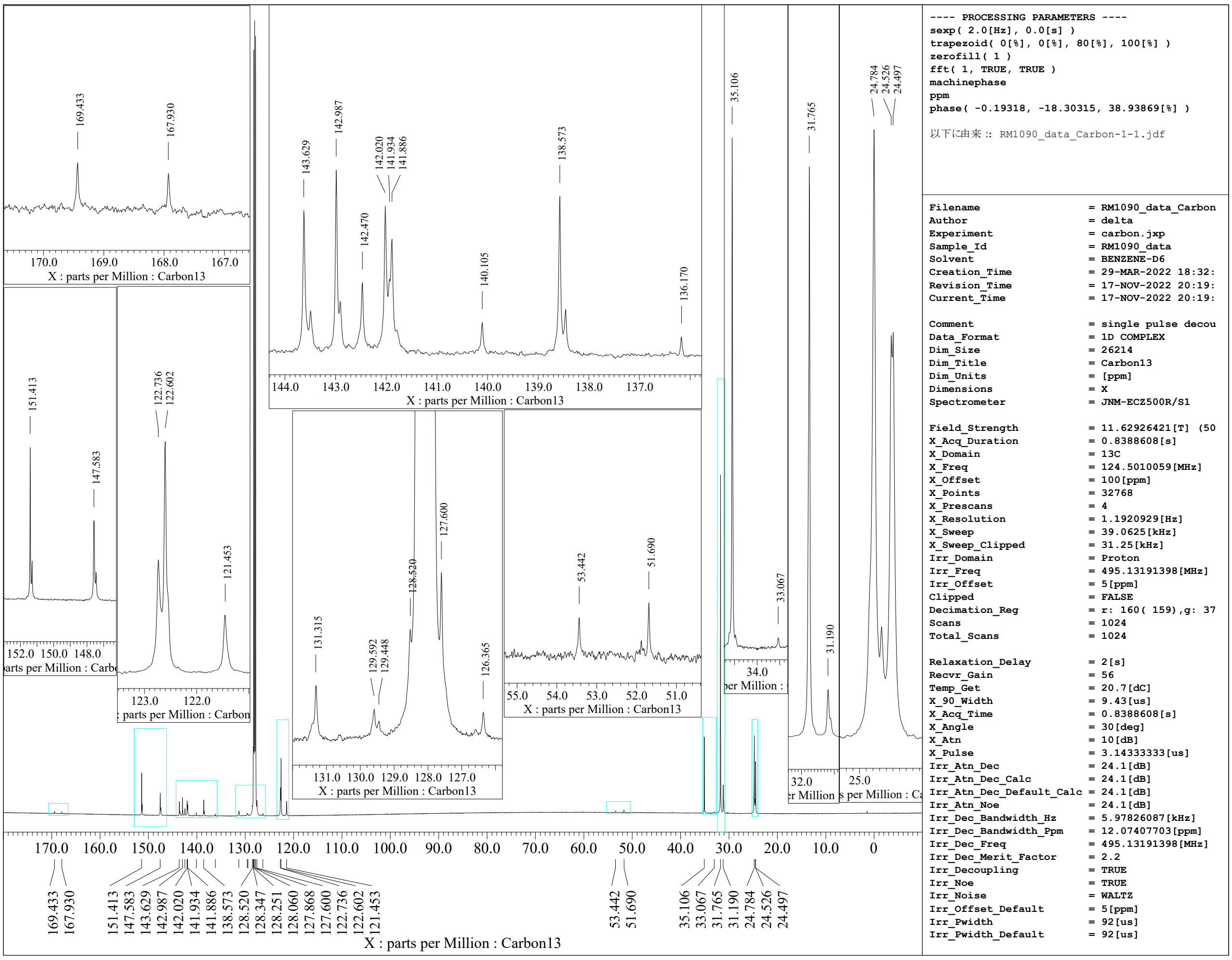

Figure S10. ^{13}C NMR (125 MHz, C_6D_6) spectrum of the mixture obtained in Scheme S9.

The compound data of **6b** were collected for the yellow crystals obtained by purification using GPLC ($CHCl_3$).

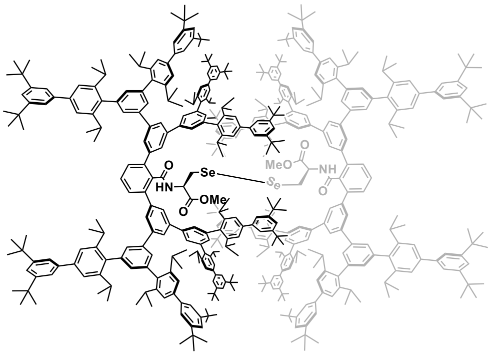

6b: yellow crystals; m.p. 242.0 °C (decomp.). **^{1}H NMR** (500 MHz, $CDCl_3$): δ 1.03-1.05 (m, 96H), 1.12-1.24 (m, 96H), 1.34 (s, 288H), 2.77-2.87 (m, 32H), 2.92-2.94 (m, 2H), 3.08-3.15 (m, 2H), 3.12 (s, 6H), 4.50-4.53 (m, 2H), 6.13 (d, J = 5.0 Hz, 2H), 7.04 (br, 8H), 7.30 (br, 32H), 7.361-7.364 (m, 32H), 7.40 (br, 16H), 7.48-7.51 (m, 4H), 7.53 (br, 16H), 7.57 -7.60 (m, 2H), 7.73 (d, J = 1.2 Hz, 8H), 7.89 (br, 4H); **^{13}C NMR** (125 MHz, $CDCl_3$): δ 24.2 (q), 24.4 (q), 29.7 (t), 30.4 (d), 31.5 (q), 34.9 (s), 51.8 (q), 52.5 (d), 121.1 (d), 121.8 (d), 122.0 (d), 126.4 (d), 126.6 (d, overlapped, d), 129.5 (d), 130.6 (d), 131.0 (d), 133.0 (s), 137.8 (s), 140.1 (s), 140.4 (s), 140.9 (s), 141.2 (s), 141.7 (s), 142.3 (s, overlapped, s), 146.8 (s), 150.7 (s), 168.1 (s), 169.3 (s); **IR** (KBr): 3035, 2961, 2905, 2867, 1744, 1682, 1594, 1476, 1472, 1362, 1346, 1247, 869 cm^{-1}; **HRMS** (ESI-TOF) m/z 3551.9448 $[M+2Na]^{2+}$ (calcd for $C_{510}H_{648}N_2Na_2O_6Se_2^{2+}$, 3551.9371).

Measurement of the ^{77}Se NMR spectrum of Sec–SeNO 2b

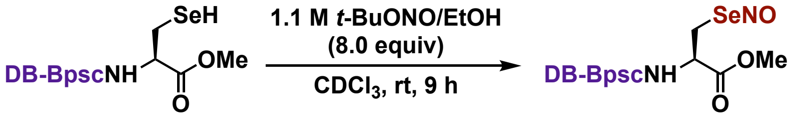

4b 2b

Measurement of the ^{77}Se NMR spectrum

Scheme S10. Observation of Sec–SeNO **2b** by ^{77}Se NMR spectroscopy.

A stock solution of *t*-BuONO (90%, 300 μL, 2.27 mmol) in dried EtOH (2.0 mL) was prepared prior to the reaction. Sec–SeH **4b** (134 mg, 0.038 mmol) was placed in a 5 mm o/d NMR tube with a J-young valve. After evacuated and backfilled with argon, freshly distilled and degassed $CDCl_3$ (0.80 mL) and the stock solution of *t*-BuONO (1.1 M, 0.27 mL, 0.30 mmol) were added. The resulting reaction mixture was degassed through freeze-pump-thaw cycles. After 9 h, a ^{77}Se NMR spectrum was recorded at room temperature (**Figure S11**). A signal of **2b** was observed at 2223 ppm. Comparison of the ^{77}Se NMR chemical shift of **2b** and those of previously reported *Se*-nitrososelenols is shown in Table S3.

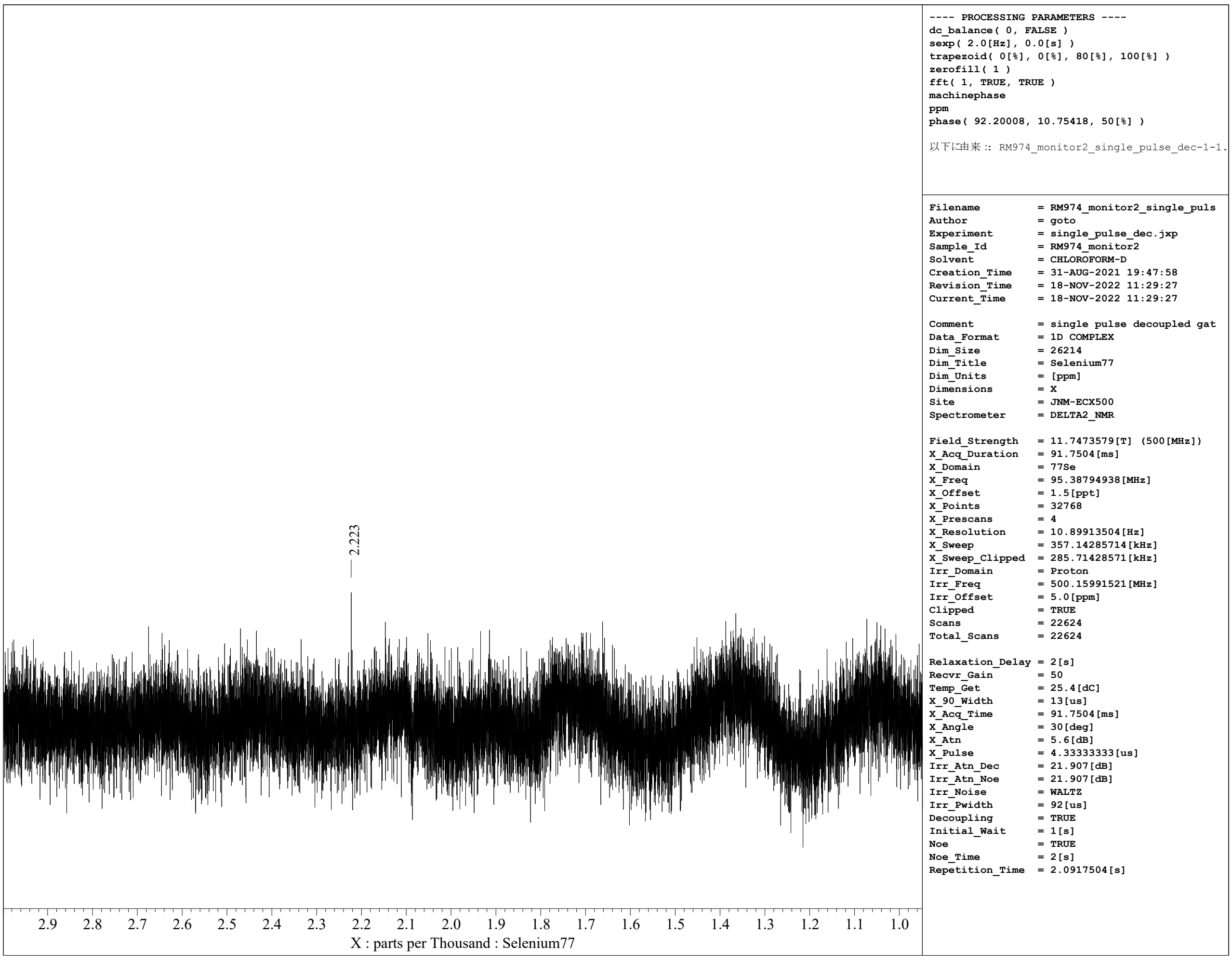

Figure S11. ^{13}Se NMR (95 MHz, $CDCl_3$) spectrum of the mixture obtained in Scheme S10.

Table S3. Comparison of the chemical shift of **2b** in the ^{77}Se NMR spectrum with those of previously reported *Se*-nitrososelenols.

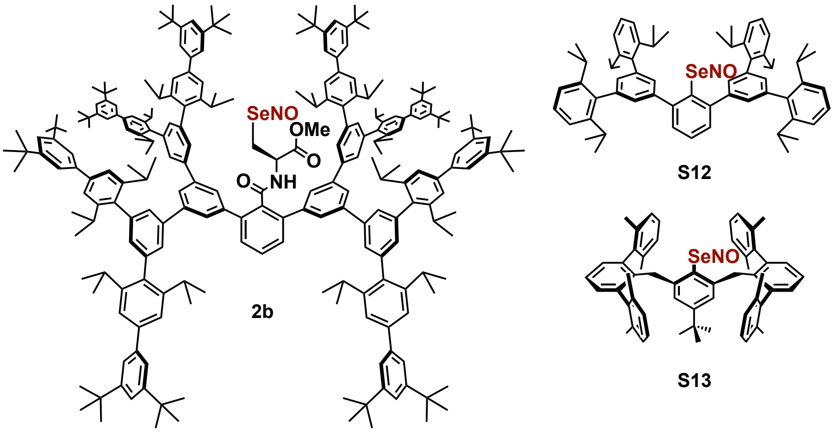

compound	δ (ppm)	solvent
**2b**	2223	$CDCl_3$
**S12**[6]	2229	$CDCl_3$
**S13**[7]	2125	$CDCl_3$

Measurement of the UV-vis spectrum of Sec–SeNO 2b

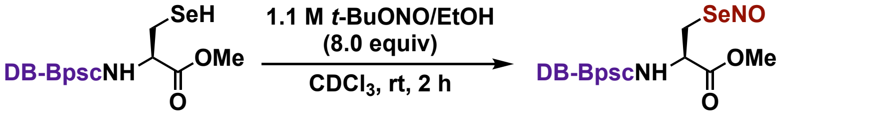

Scheme S11. Measurement of the UV-vis spectrum of Sec–SeNO **2b**.

A stock solution of *t*-BuONO (90%, 300 μL, 2.27 mmol) in dried EtOH (2.00 mL) was prepared prior to the reaction. Sec–SeH **4b** (28.6 mg, 8.10 μmol) was placed in a 5 mm o/d NMR tube with a J-young valve. After evacuated and backfilled with argon, distilled and degassed $CDCl_3$ (0.60 mL) and the stock solution of *t*-BuONO (1.14 M, 57 μL, 64.8 μmol) were added. The resulting reaction mixture was degassed through freeze-pump-thaw cycles, and the tube was then flushed with argon. After 2 h, the entire amount of the resulting reddish-orange mixture was transferred to a quartz cell in a glove box (N_2), and then $CDCl_3$ (0.10 mL) was added to the solution. A UV-vis spectrum was recorded at 298 K (**Figure S12**). The assignments of the absorptions at 495 nm and 647 nm were based on the reported DFT calculation for C_6H_5SeNO at the B3LYP/6-311+G(2d)//B3LYP/6-31G(d) level,[6] which showed two characteristic bands at 495 nm (n–π^* transition) and 711 nm (π–π^* transition). Comparison of the absorption maximum of **2b** with those of previously reported *Se*-nitrososelenols is shown in Table S4.

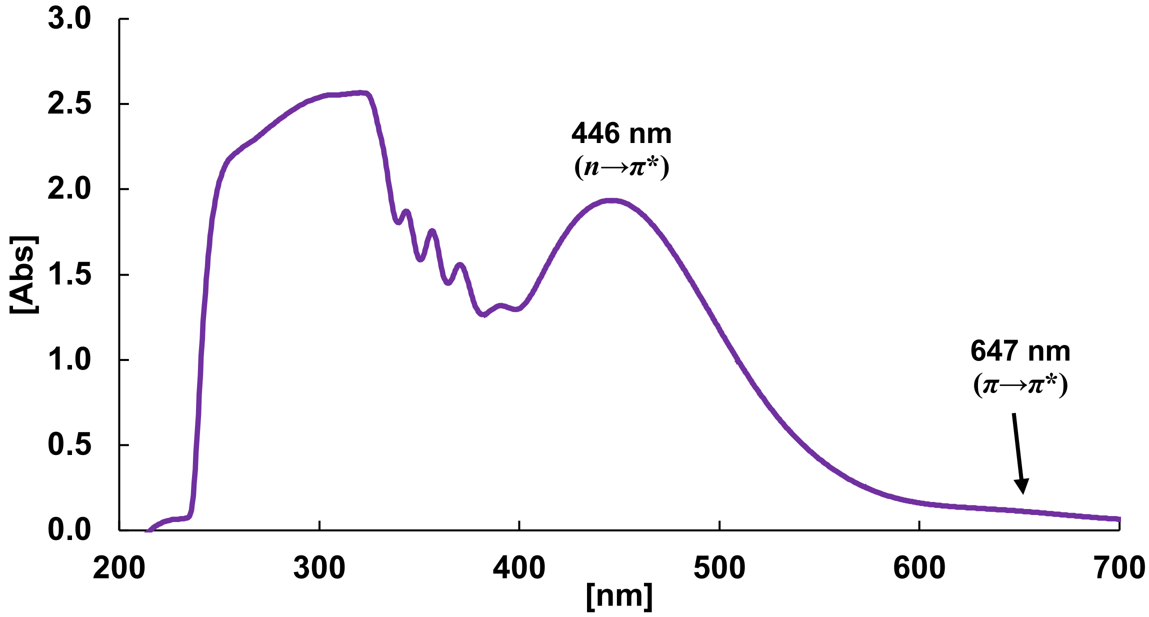

Figure S12. UV-vis ($CDCl_3$, 298 K) spectrum of the mixture obtained in Scheme S11.

Table S4. Comparison of the absorption maximum of **2b** in UV-vis NMR spectrum with those of previously reported *Se*-nitrososelenols.

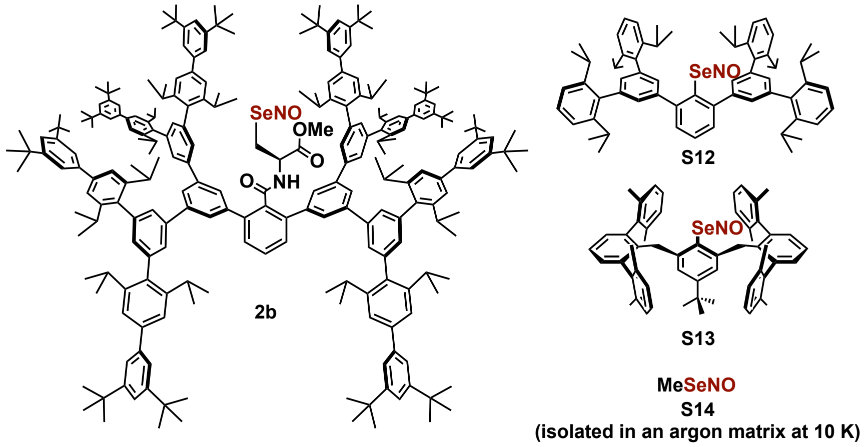

compound	λ_{max} (nm)	media
**2b**	446 (ε ca. 167)	$CDCl_3$
**S12**[6]	485 (ε 150)	$CHCl_3$
**S13**[7]	467 (ε 160)	$CHCl_3$
**S14**[8]	440	argon, 10 K

Investigation of transnitrosation from GSNO to Sec–SeH 4b

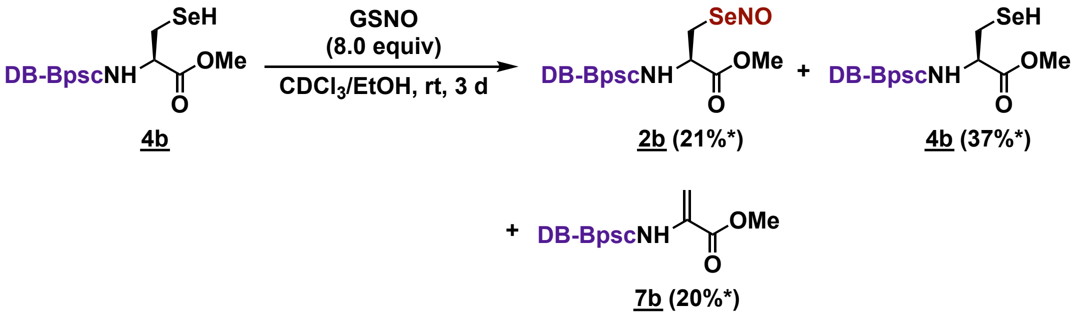

Scheme S12. Investigation of transnitrosation from GSNO to Sec–SeH **4b**.

Sec–SeH **4b** (20.2 mg, 5.72 μmol) and GSNO (15.4 mg, 45.8 μmol), which was prepared by the reported procedure,[9] were placed in a 5 mm o/d NMR tube with a J-Young valve. After evacuated and backfilled with argon, freshly distilled and degassed $CDCl_3$ (0.60 mL) and EtOH (20 μL) were added. The resulting reaction mixture was degassed through freeze-pump-thaw cycles, and the tube was then flushed with argon. The ^{1}H NMR spectra at selected times were recorded. After 3 days, the ^{1}H NMR yields of Sec–SeNO **2b**, the starting material **4b**, and the corresponding dehydroalanine **7b** were estimated to be 21%, 37%, and 20%, respectively. The signals of **7b** in the ^{1}H NMR spectrum were in agreement with those of the isolated sample prepared by another route (**Scheme S15**).

Investigation of the reactivity of Sec–SeNO 2b

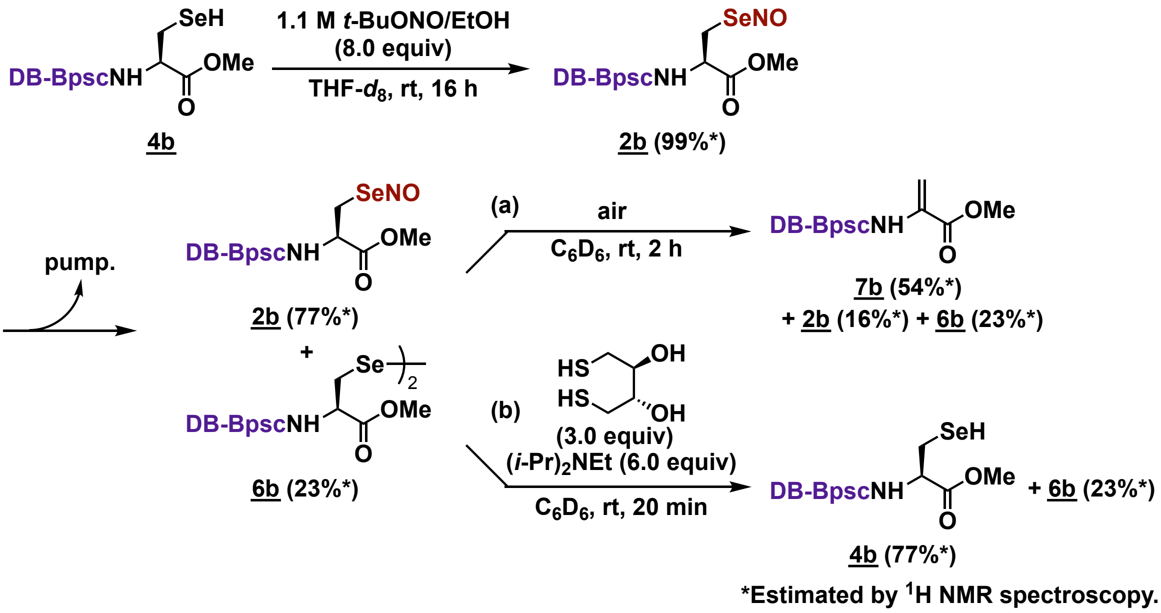

Scheme S13. Investigation of reactivity of Sec–SeNO **2b**.

A stock solution of *t*-BuONO (90%, 300 µL, 2.27 mmol) in dried EtOH (2.0 mL) was prepared prior to the reaction. Sec–SeH **4b** (34.9 mg, 9.88 µmol) was placed in a 5 mm o/d NMR tube with a J-young valve. After evacuated and backfilled with argon, freshly distilled and degassed THF-d_8 (0.70 mL) and the stock solution of *t*-BuONO (1.1 M, 69 µL, 79 µmol) were added. The resulting reaction mixture was degassed through freeze-pump-thaw cycles, and the tube was then flushed with argon. The ^{1}H NMR spectra at selected times were recorded. After 16 h, the formation of Sec–SeNO **2b** in 98% NMR yield was observed by ^{1}H NMR spectroscopy. Then, the solvent was removed in a glove box to give orange crystals (**2b**:**6b** = 77:23). The entire amount of the obtained crystals was dissolved in C_6D_6 (1.8 mL) and the solution was divided into three equal parts, each of which was placed to a 5 mm o/d NMR tube with a J-Young valve. The resulting three samples were used for the following experiments.

(a) Stability of Sec–SeNO **2b** in air.

The sample was left in air at room temperature, and the reaction was monitored by ^{1}H NMR spectroscopy. After 2 h, the NMR yields of the corresponding dehydroalanine **7b**, the remained Sec–SeNO **2b**, and diselenide **6b** were estimated to be 54%, 16%, and 23%, respectively.

(b) Reaction of Sec–SeNO **2b** with dithiothreitol (DTT).

To a solution in a J-young NMR tube were added DTT (1.5 mg, 99 µmol), (*i*-Pr)$_2$NEt (3.4 µL, 20 µmol), and the tube was carefully sealed. The sample was left at room temperature, and the reaction was monitored by ^{1}H NMR spectroscopy. After 20 min, Sec–SeH **4b** was formed in quantitative conversion yield.

Investigation of the reactivity of Sec–SeNO 2b toward D_2O

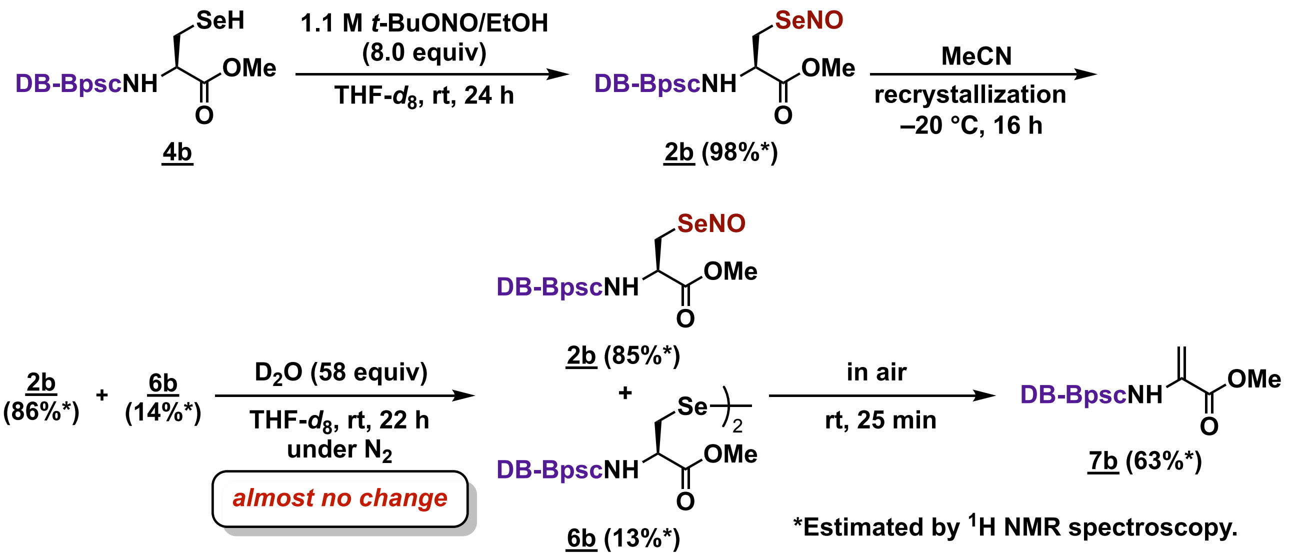

Scheme S14. Reaction of Sec–SeNO **2b** with D_2O under inert atmosphere or in air.

A stock solution of *t*-BuONO (90%, 300 μL, 2.27 mmol) in dried EtOH (2.0 mL) and D_2O (10 μL, 0.55 mmol) in THF-d_8 (0.50 mL) were prepared prior to the reaction. Sec–SeH **4b** (23.6 mg, 6.68 μmol) was placed in a 5 mm o/d NMR tube with a J-young valve. After evacuated and backfilled with argon, degassed (freeze-pump-thaw cycles) THF-d_8 (0.60 mL) and the stock solution of *t*-BuONO (1.1 M, 47 μL, 53 μmol) were added in a glove box filled with argon. After 24 h, the formation of Sec–SeNO **2b** in 98% NMR yield was observed by ^{1}H NMR spectroscopy. Then, in the glove box, all amount of the resulting mixture was poured into cold MeCN (–20 °C) and the resulting mixture was stored in refrigerator (–20 °C) for 16 h. The filtration of the resulting orange suspension to give orange powder (**2b**:**6b** = 86:14). In the glove box (argon), a part of the obtained crystals (3.3 mg, 0.80 μmol for **2b**) was dissolved in THF-d_8 (0.55 mL) in a 5 mm o/d NMR tube with a J-young valve and the resulting sample was transferred in a glove box (N_2). After the stock solution of D_2O in THF-d_8 (1.1 M, 43 μL, 46 μmol) was added to the sample tube, the ^{1}H NMR spectra at selected times were recorded. Almost no change was observed after 22 h; Sec–SeNO **2b** and the corresponding diselenide **6b** were remained in 85% and 13% NMR yields, respectively. Then, the J-young valve was opened in air and the ^{1}H NMR spectra at selected times were recorded. The immediate decompose of **2b** was observed; the formation of the corresponding dehydroalanine **7b** in 63% NMR yield after 25 min.

Reaction of Sec–SeNO 2b with a cysteine thiol

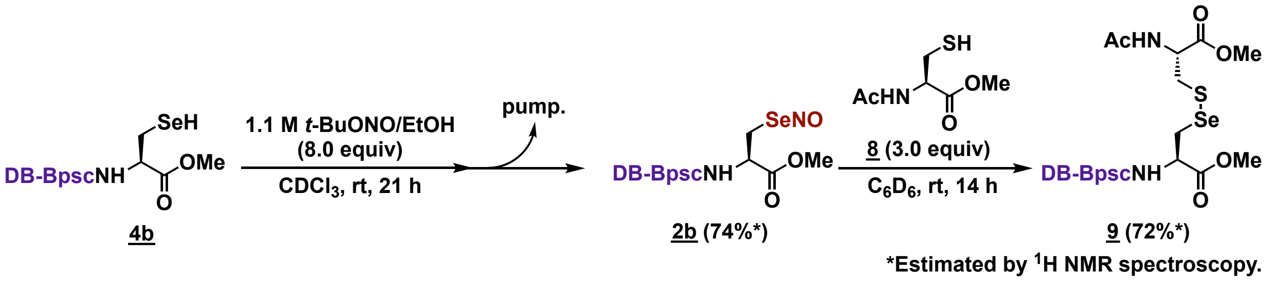

Scheme S15. Reaction of Sec–SeNO **2b** with cysteine thiol **8**.

A stock solution of *t*-BuONO (90%, 300 μL, 2.27 mmol) in dried EtOH (2.0 mL) was prepared prior to the reaction. Sec–SeH **4b** (12.9 mg, 3.65 μmol) was placed in a 5 mm o/d NMR tube with a J-Young valve. After evacuated and backfilled with argon, freshly distilled and degassed $CDCl_3$ (0.70 mL) and the stock solution of *t*-BuONO (1.14 M, 26 μL, 29 μmol) were added. The resulting reaction mixture was degassed through freeze-pump-thaw cycles, and the tube was then flushed with argon. The ^{1}H NMR spectra at selected times were recorded. After 21 h, the solvent was removed in a glove box to give orange crystals, which was then dissolved in C_6D_6 (0.60 mL). The ^{1}H NMR yields of **2b** and **6b** in the obtained crystals were estimated to be 74% and 26%, respectively. To the solution of the mixture in a J-Young NMR tube, *N*-acetyl cysteine methyl ester (**8**; 1.9 mg, 11 μmol) was added in a glove box (Ar). After 14 h, the formation of the corresponding selenenyl sulfide **9** was observed in 97% conversion yield as estimated by the ^{1}H NMR spectroscopy. The signals of **9** in the ^{1}H NMR spectrum were in agreement with those of the isolated sample prepared by another route (**Scheme S17**).

Identification of dehydroalanine 7b

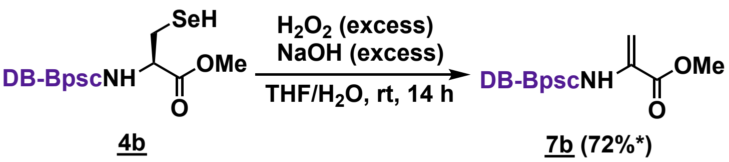

Scheme S16. Identification of dehydroalanine **7b**.

Sec–SeH **4b** (64.8 mg, 18.4 μmol) was placed in a 10 mL J-Young tube. After evacuated and backfilled with argon, THF (4.0 mL), H_2O_2 (30%, 0.3 mL, 3 mmol) and then 2 M aq. NaOH (0.1 mL, 0.2 mmol) were added. The resulting reaction mixture was stirred at room temperature for 14 h before saturated aq. Na_2SO_3 (2 mL) was added. The two layers were separated and the aqueous layer was extracted with $CHCl_3$ (3×8 mL). The combined organic layer was washed with brine, dried over Na_2SO_4, and filtered. The filtrate was concentrated in vacuo and the crude product was purified by trituration with CH_3CN to give **7b** as colorless crystals. Yield 45.6 mg (13.2 μmol, 72%).

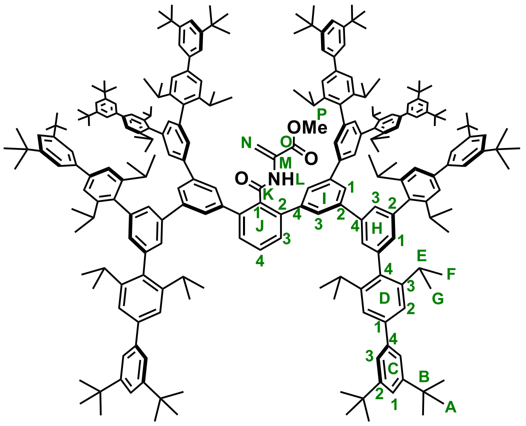

7b: colorless crystals; m.p. 257.0-258.0 °C. **^{1}H NMR** (500 MHz, $CDCl_3$): δ 1.09 (d, J = 7.0 Hz, 48H, **F** or **G**), 1.16 (d, J = 6.5 Hz, 48H, **F** or **G**), 1.38 (s, 144H, **A**), 2.82 (septet, J = 7.0 Hz, 16H, **E**), 3.11 (s, 3H, **P**), 5.20 (s, 1H, **N$_A$**), 6.29 (s, 1H, **N$_B$**), 7.07 (br, 4H, **H1**), 7.33 (br, 16H, **D2**), 7.39 (d, J = 2.0 Hz, 16H, **C3**), 7.43 (t, J = 2.0 Hz, 8H, **C1**), 7.48 (d, J = 1.0 Hz, 8H, **H3**), 7.57 (br, 3H, **J3** and **J4**), 7.73 (d, J = 1.0 Hz, 4H, **I3**), 7.85 (br s, 1H, **L**), 7.86 (br, 2H, **I1**); **^{13}C NMR** (125 MHz, $CDCl_3$): δ 24.2, 24.3 (q, **F** and **G**), 30.5 (d, **E**), 31.6 (q, **A**), 34.9 (s, **B**), 52.4 (q, **P**), 108.1 (t, **N**), 121.1 (d, **C3**), 121.9 (d, **D2**, overlapped, d, **C1**), 125.7 (d, **I1**), 126.58 (d, **I3**), 126.65 (d, **H3**), 129.6 (d, **J3**), 130.0 (d, **J4**), 130.5 (d, **H1**, overlapped, s, **M**), 135.4 (s, **J1**), 137.9 (s, **D4**), 140.2 (s, **J2**), 140.4 (s, **I2**), 140.9 (s, **D1**), 141.1 (s, **I4**), 141.8 (s, **H2**), 141.9 (s, **H4**), 142.2 (s, **C4**), 146.9 (s, **D3**), 150.9 (s, **C2**), 163.4 (s, **O**), 167.9 (s, **K**); **IR** (KBr): 3387, 3035, 2961, 2904, 2867, 1724, 1684, 1594, 1463, 1393, 1362, 1314, 1247, 1216, 909, 870, 757 cm^{-1}; **HRMS** (ESI-TOF) m/z 3470.5128 $[M+Na]^+$ (calcd for $C_{255}H_{323}NNaO_3$, $[M+Na]^+$, 3470.5045).

Identification of selenenyl sulfide 9

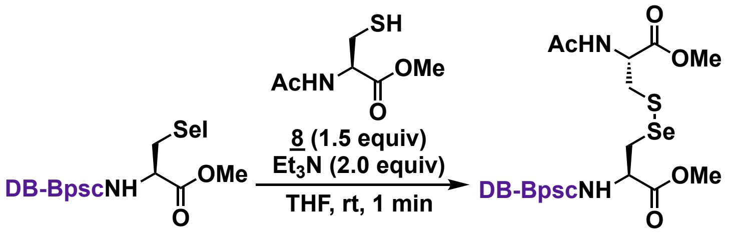

Scheme S17. Identification of selenenyl sulfide **9**.

Sec–SeI **5b** (64.7 mg, 17.7 μmol) was placed in a 30 mL two-necked flask. After evacuated and backfilled with argon, THF (1.0 mL), cysteine thiol **8** (4.7 mg, 27 μmol) and then Et_3N (5.0 mL, 35 μmol) were added. The resulting solution was stirred at room temperature for 1 min before brine was added. The two layers were separated and the aqueous layer was extracted with CH_2Cl_2 (3×4 mL). The combined organic layer was washed with brine, dried over Na_2SO_4, and filtered. The filtrate was concentrated in vacuo and the crude product was purified by trituration in CH_3CN to give **9** as colorless crystals. Yield 46.8 mg (12.6 μmol, 71%).

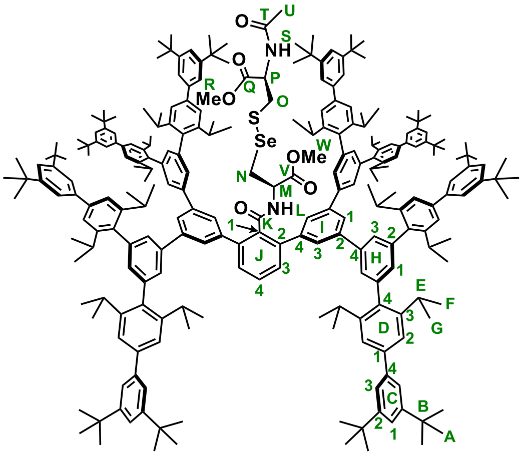

9: colorless crystals; m.p. 234.0-235.0 °C. ^{1}**H NMR** (500 MHz, $CDCl_3$, 50 °C): δ 1.13-1.14 (m, 48H, **F** and **G**), 1.20-1.22 (m, 48H, **F** and **G**), 1.40 (s, 144H, **A**), 1.77 (s, 3H, **U**), 2.43 (br dd, *J* = 14.0, 6.0 Hz, 1H, $\mathbf{N_A}$), 2.83-2.90 (m, 17H, **E** and $\mathbf{O_A}$), 3.05 (dd, *J* = 13.5, 2.5 Hz, 1H, $\mathbf{N_B}$), 3.09 (dd, *J* = 13.5, 5.0 Hz, 1H, $\mathbf{O_B}$), 3.17 (s, 3H, **W**), 3.47 (s, 3H, **R**), 4.50-4.54 (m, 1H, **P**), 4.79-4.82 (m, 1H, **M**), 6.20 (d, *J* = 7.0 Hz, 1H, **S**), 6.55 (d, *J* = 7.5 Hz, 1H, **L**), 7.11 (br, 4H, **H1**), 7.36 (br, 16H, **C3**), 7.41 (br, 16H, **D2**), 7.44 (br, 8H, **C1**), 7.48-7.49 (m, 3H, **J3** and **J4**), 7.53 (br, 8H, **H3**), 7.75 (br, 4H, **I3**), 7.87 (br, 2H, **I1**); ^{13}**C NMR** (125 MHz, $CDCl_3$): δ 22.6 (q, **U**), 24.2, 24.4 (q, **F** and **G**), 30.52, 30.55 (d, **E**), 31.5 (q, **A**), 32.2 (t, **N**), 34.9 (s, **B**), 41.2 (t, **O**), 50.0 (d, **M**), 51.9 (d, **P**), 52.2 (q, **R**), 52.3 (q, **W**), 121.2 (d, **C1**), 121.9 (d, **D2**), 122.0 (d, **C3**), 126.0 (d, **I1**), 126.7 (d, **H3**), 126.8 (d, **I3**), 129.5 (d, **J4**), 130.1 (d, **J3**), 130.7 (d, **H1**), 134.3 (s, **J1**), 137.8 (s, **D4**), 140.2 (s, **J2**), 140.4 (s, **I2**), 141.0 (s, **D1**), 141.7 (s, **H2**, overlapped, s, **I4**, and s, **H4**), 142.4 (s, **C4**), 146.9 (s, **D3**), 150.9 (s, **C2**), 168.1 (s, **K**), 169.7 (s, **T**), 170.1 (s, **Q**), 170.5 (s, **V**); **IR** (KBr): 3036, 2960, 2904, 2867, 1748, 1683, 1594, 1476, 1463, 1362, 1247, 869, 691 cm^{-1}; **HRMS** (ESI-TOF) *m/z* 3727.4745 $[M+Na]^+$ (calcd for $C_{261}H_{334}N_2NaO_6SSe$, 3727.4746).

2. X-ray crystallographic analysis

X-ray crystallographic analysis of 5b

Single crystals of 2(**5b**)·16.7($CHCl_3$) were grown in their $CHCl_3$-benzene-EtOH solution. A purple crystal of 2(**5b**)·16.7($CHCl_3$) was mounted on a loop. All measurements were made on a Rigaku/Synergy CCD with VariMax Cu with graphite monochromated Cu-Kα radiation (λ = 1.54184 Å) at –150 °C. Crystallographic and experimental

data are listed in **Table S5**. The structures were solved and refined against all F_2 values using Shelx-2018 implemented through Olex2 v1.5. The non-hydrogen atoms were refined anisotropically, except for the minor components of the disordered *tert*-butyl groups and solvents. The hydrogen atoms were idealized by using the riding models. Attempt to sensibly model the solvent molecules (probably five chloroform molecules which were used for final crystallization) was unsuccessful because of diffuse electron density (disordered) corresponding to them and limited data quality. So, the solvent mask (similar to PLATON_SQUEEZE) was applied using Olex2 to remove those electron densities in the final model. The solvent accessible volume was found to be 2220.0 Å^3. The number of the electrons found in solvent accessible void is 309.9 e$^-$, which corresponds to approximately five chloroform molecules per unit cell. The slight variation in void electron count can be the result of limited data quality.

Table S5. Crystal data and structure refinement for 2(**5b**)·16.7($CHCl_3$).

Empirical formula	2($C_{255}H_{324}INO_3Se$)·16.7($CHCl_3$)	
Formula weight	9307.43	
Temperature	123(2) K	
wavelength	1.54184 Å	
Crystal system	Triclinic	
Space group	*P*1	
Unit cell dimensions	*a* = 21.3222(2) Å	*a* = 93.5740(10)°
	b = 22.3671(2) Å	*b* = 107.1930(10)°
	c = 35.0156(4) Å	*g* = 114.4860(10)°
Volume	14186.5(3) Å^3	
Z	1	
D_{calc}	1.089 g/cm^3	
Absorption coefficient	3.560 mm^{-1}	
F(000)	4913.0	
Crystal size	0.25 x 0.18 x 0.12 mm^3	
Theta range for data collection	2.329 to 76.697°	
Index ranges	$-26 \leq h \leq 26$, $-27 \leq k \leq 27$, $-43 \leq l \leq 43$	
Reflections collected	407605	
Independent reflections	104558 [R_{int} = 0.1081]	
Max. and min. transmission	0.629 and 1.000	
Completeness to theta = 135.4°	99.9%	
data / restraints / parameters	104558 / 138 / 5485	
Goodness-of-fit on F^2	1.171	
Final R induces (I>2σ (I))	$R1$ = 0.1130, w$R2$ = 0.3009	
R induces (all data)	$R1$ = 0.1408, w$R2$ = 0.3239	
Largest diff. peak and hole	1.88 and −0.82 e. Å^{-3}	
Flack parameter	0.090(4)	

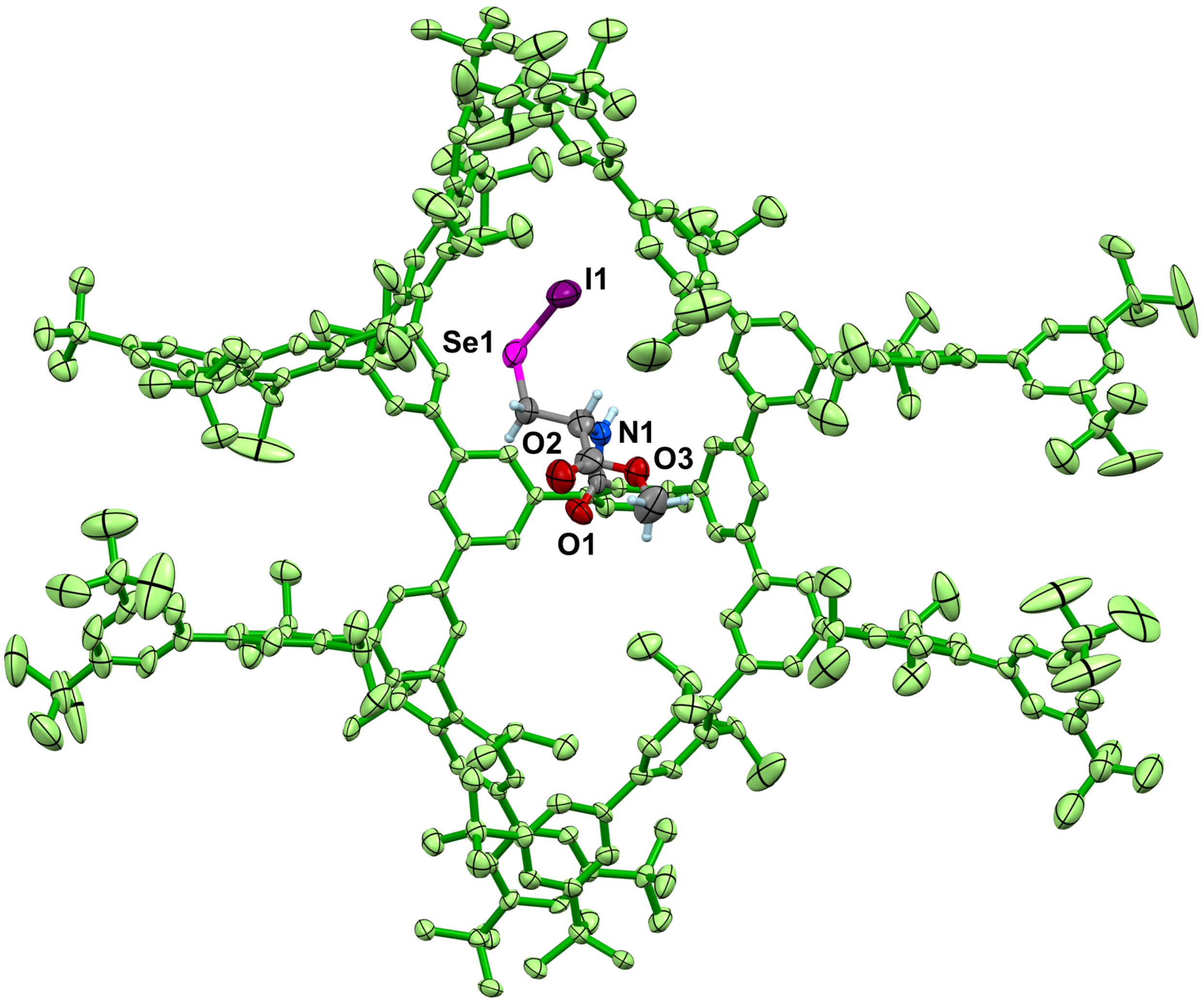

Figure S13. Selected molecular structure of one of the two independent molecules of **5b** with thermal ellipsoids at 50% probability (CCDC 2251497). Only the position with higher occupancy of the disordered *tert*-butyl groups in the DB-Bpsc groups and the disordered Se–I bond in the selenocysteine moiety are shown. Hydrogen atoms of the DB-Bpsc group are omitted for clarity.

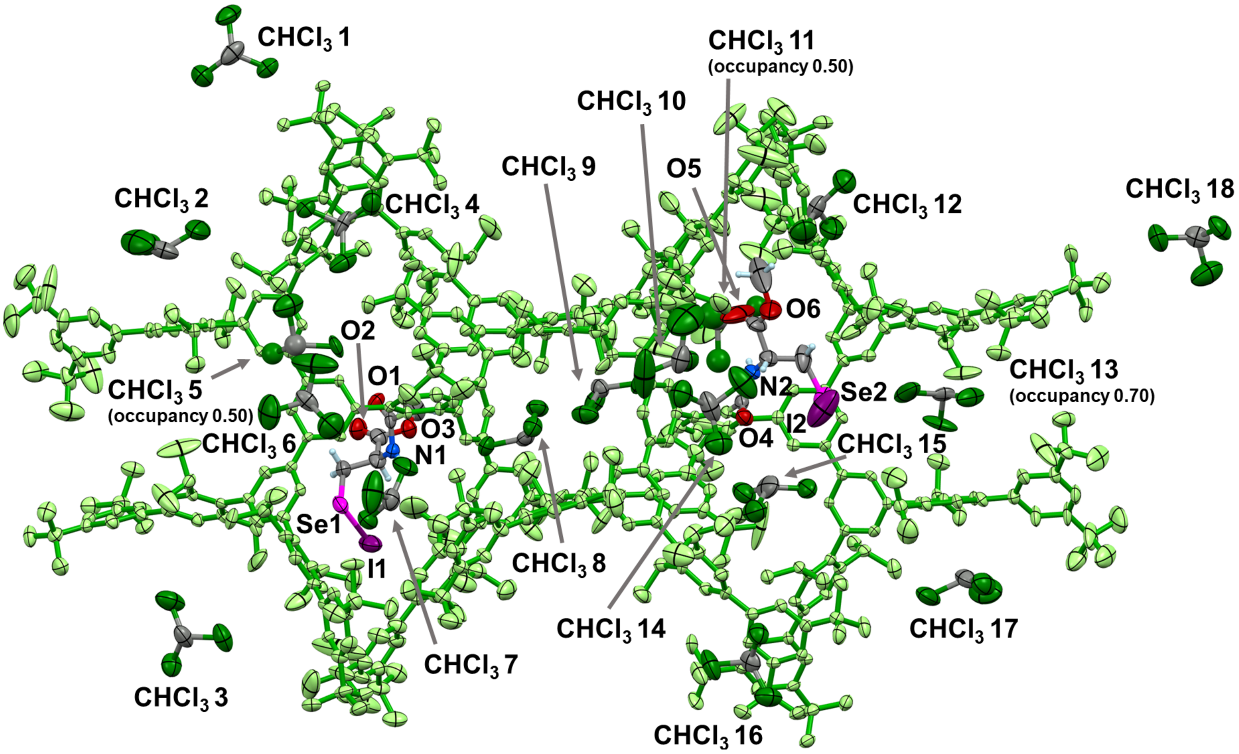

Figure S14. Molecular structure of **5b** with thermal ellipsoids at 50% probability (CCDC 2251497). Only the position with higher occupancy of the disordered *tert*-butyl groups in the DB-Bpsc group and the disordered Se–I bonds in the selenocysteine moiety are shown. Hydrogen atoms of the DB-Bpsc groups and solvents are omitted for clarity.

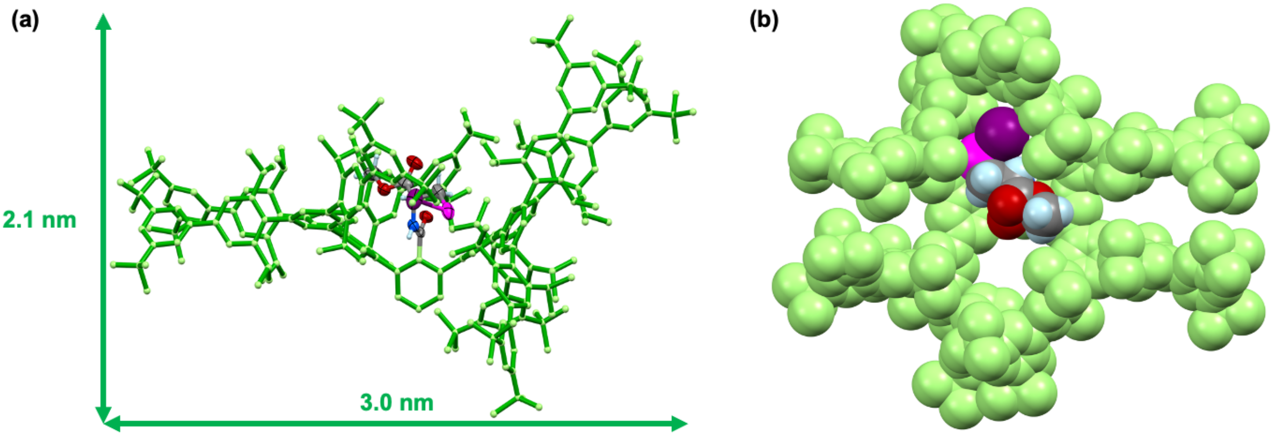

Figure S15. Selected molecular structure of one of the two independent molecules of **5b** with thermal ellipsoids at 50% probability (CCDC 2251497). (a) Drawn by ball and stick style. (b) Drawn by CPK style. Only the position with higher occupancy of the disordered *tert*-butyl groups in the DB-Bpsc group and the disordered Se–I bonds in the selenocysteine moiety are shown. Hydrogen atoms of the DB-Bpsc groups are omitted for clarity.

3. NMR spectra

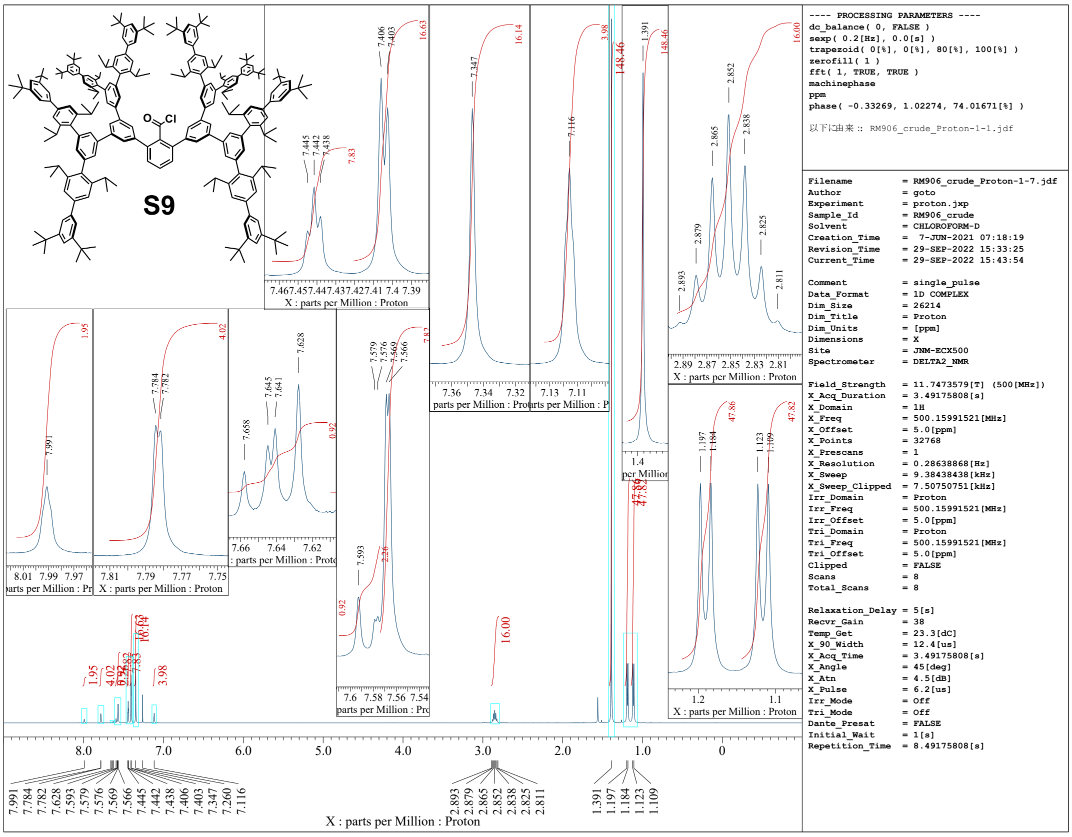

Figure S16. ^{1}H NMR (500 MHz, $CDCl_3$) spectrum of **S9**.

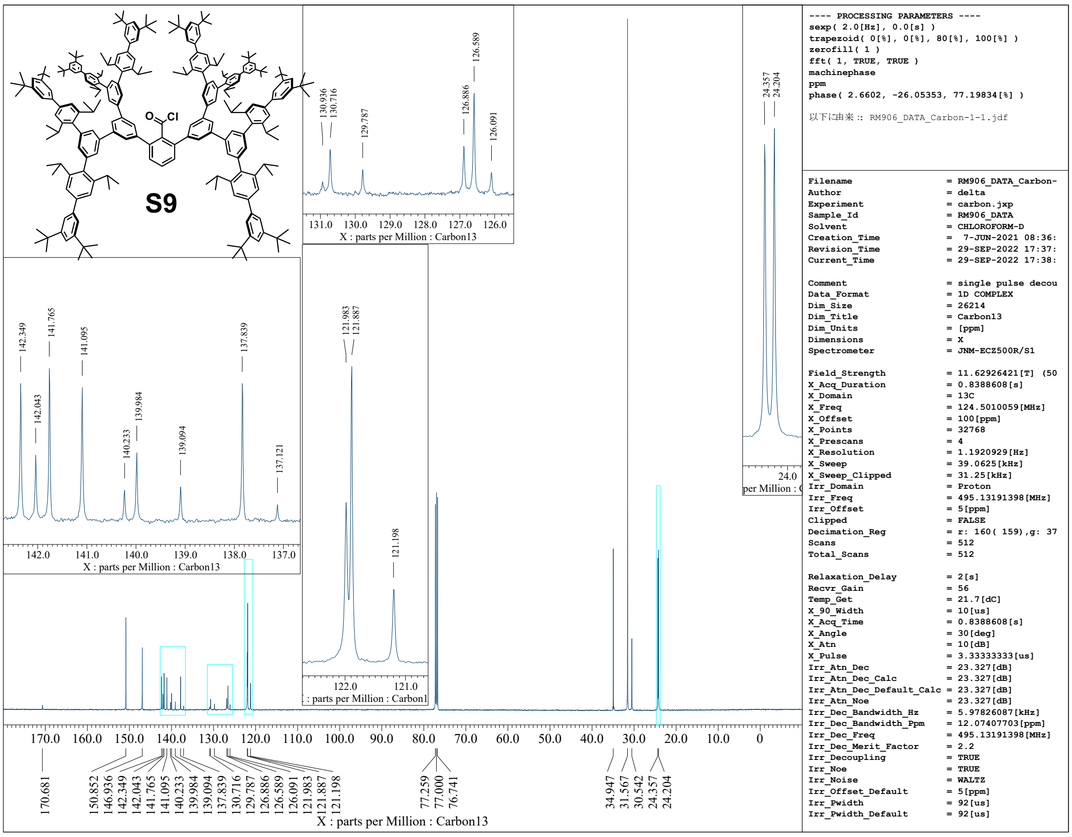

Figure S17. ^{13}C NMR (125 MHz, $CDCl_3$) spectrum of **S9**.

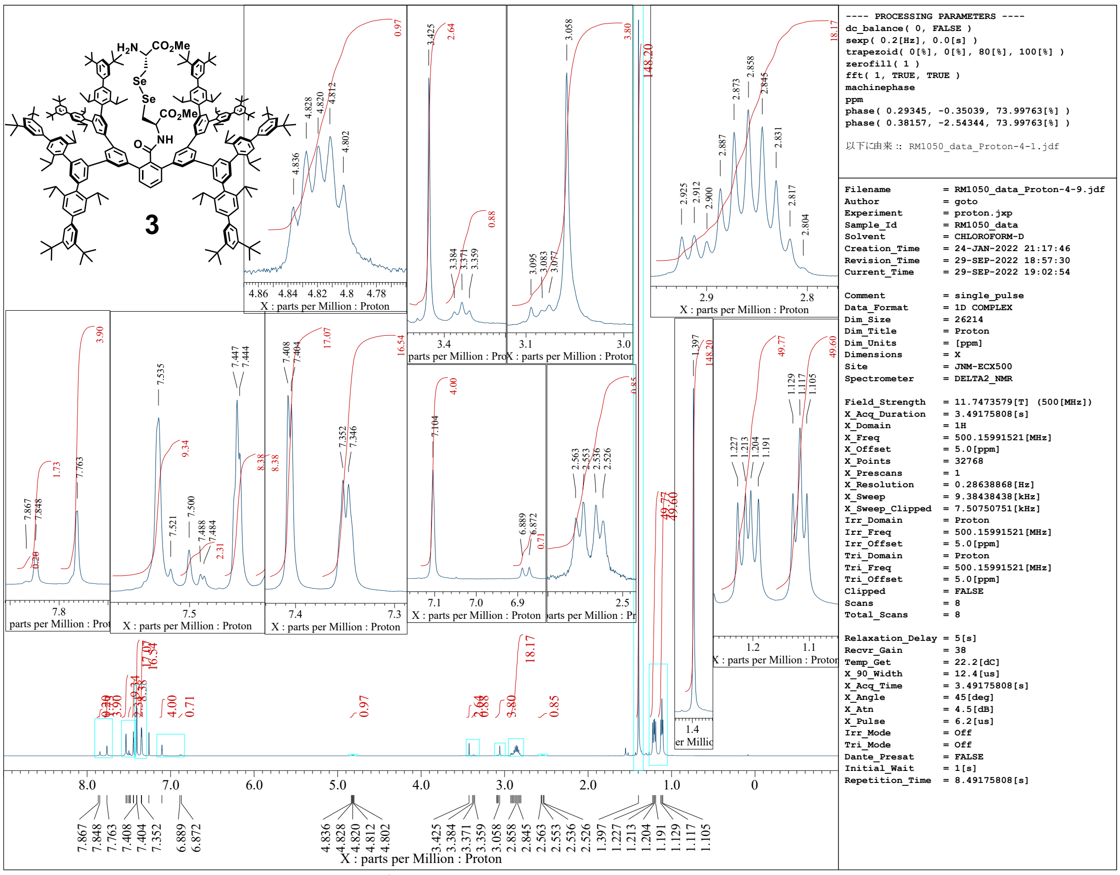

Figure S18. ^{1}H NMR (500 MHz, $CDCl_3$) spectrum of **3**.

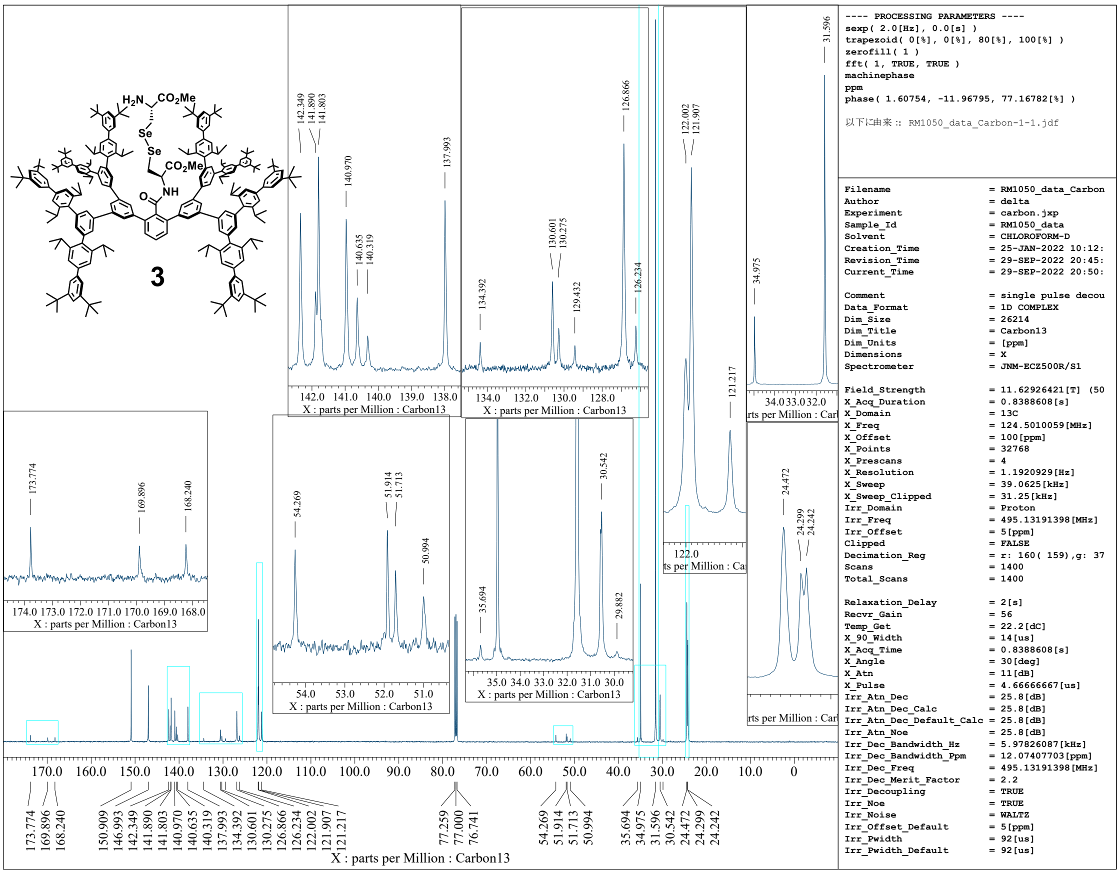

Figure S19. ^{13}C NMR (125 MHz, $CDCl_3$) spectrum of **3**.

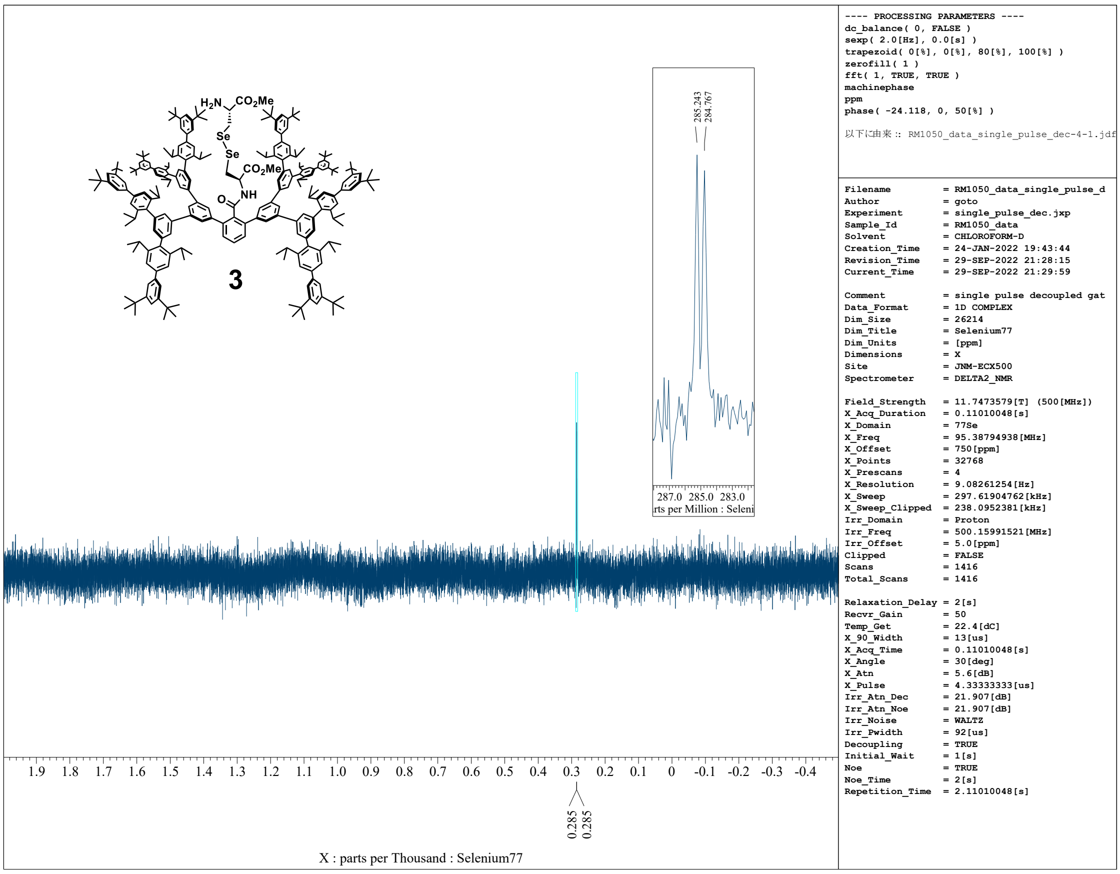

Figure S20. ^{77}Se NMR (95 MHz, $CDCl_3$) spectrum of **3**.

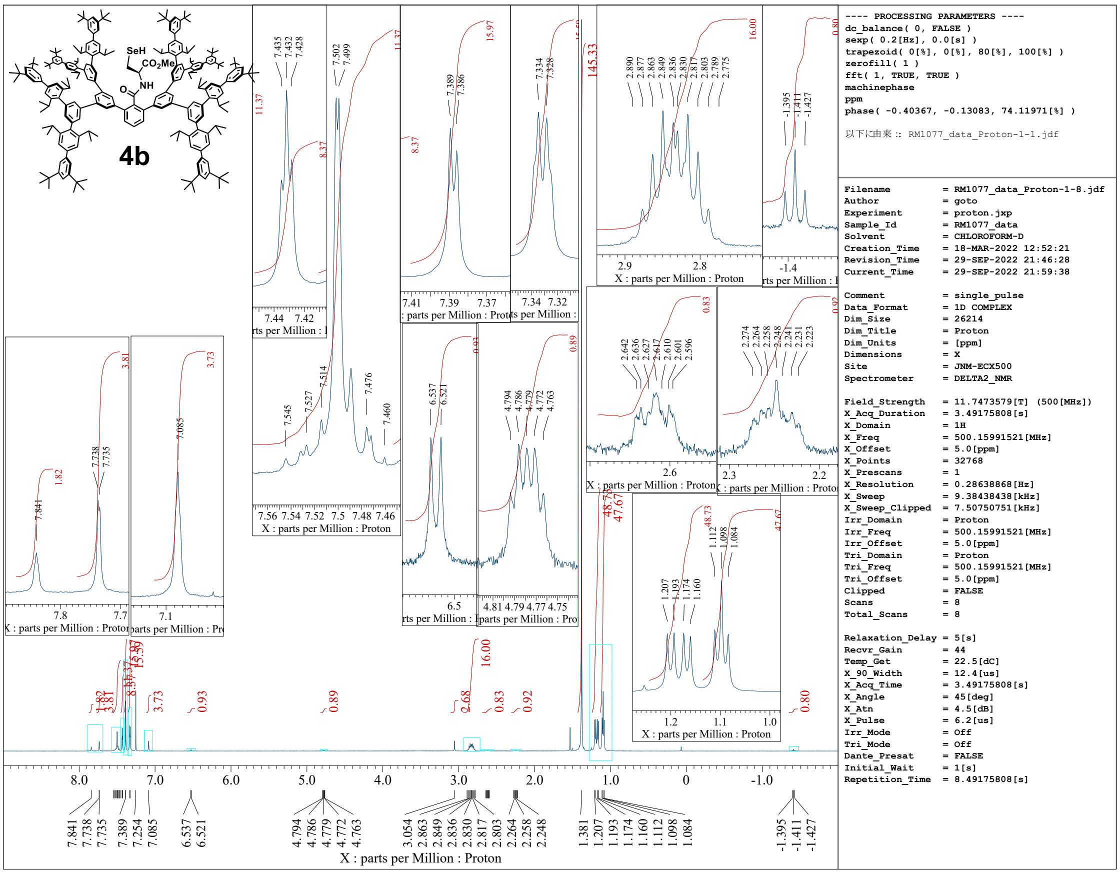

Figure S21. ^{1}H NMR (500 MHz, $CDCl_3$) spectrum of **4b**.

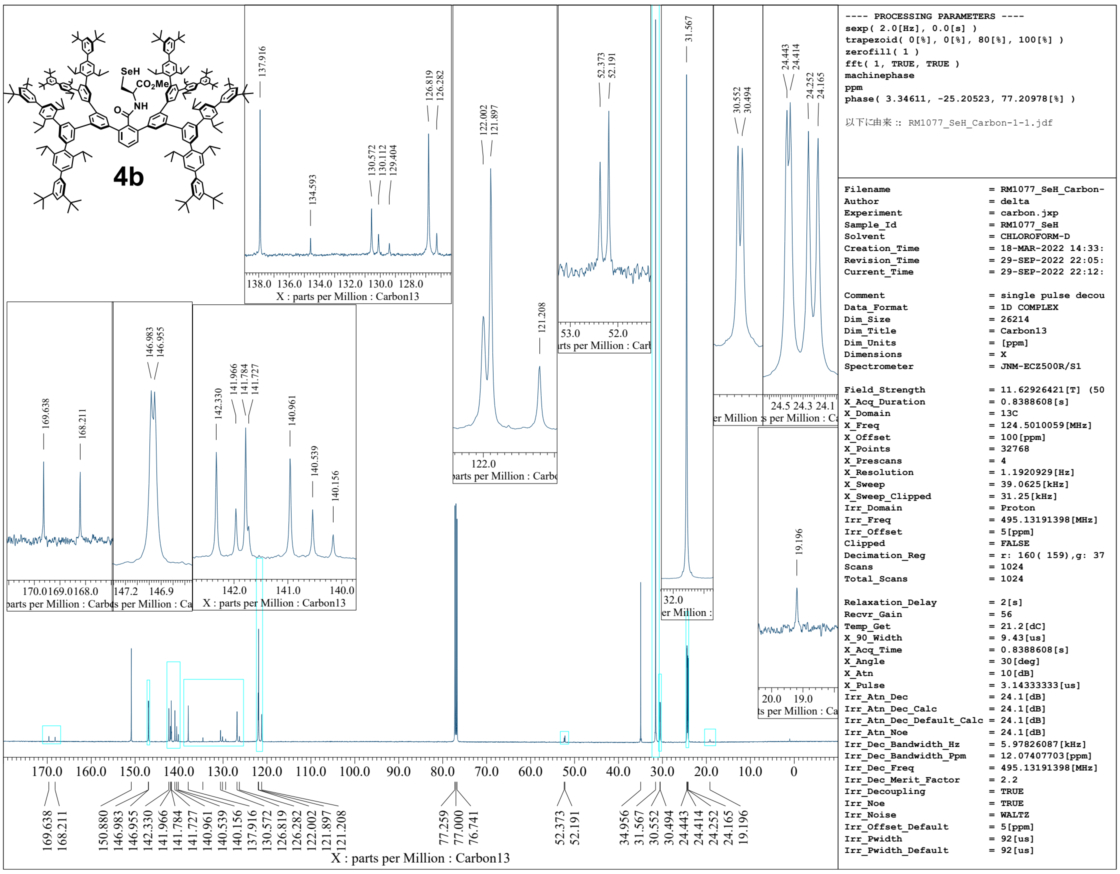

Figure S22. ^{13}C NMR (125 MHz, $CDCl_3$) spectrum of **4b**.

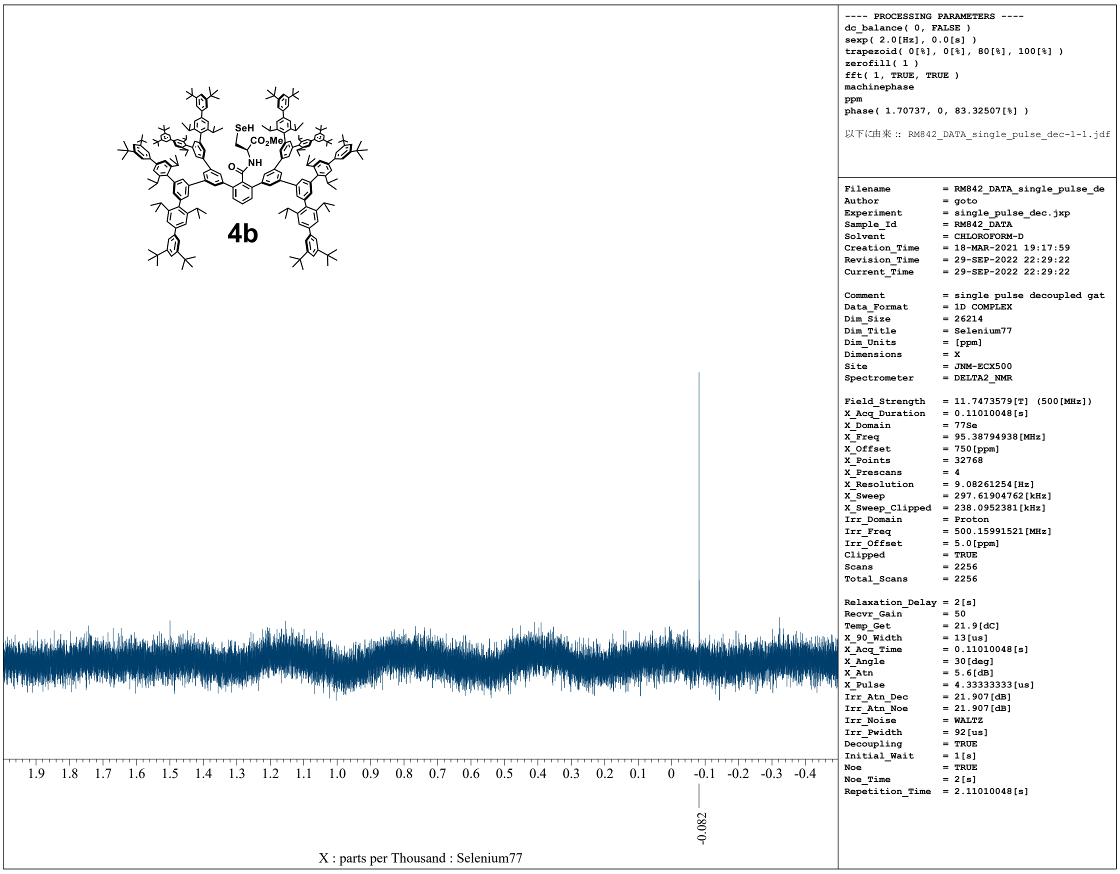

Figure S23. ^{77}Se NMR (95 MHz, $CDCl_3$) spectrum of **4b**.

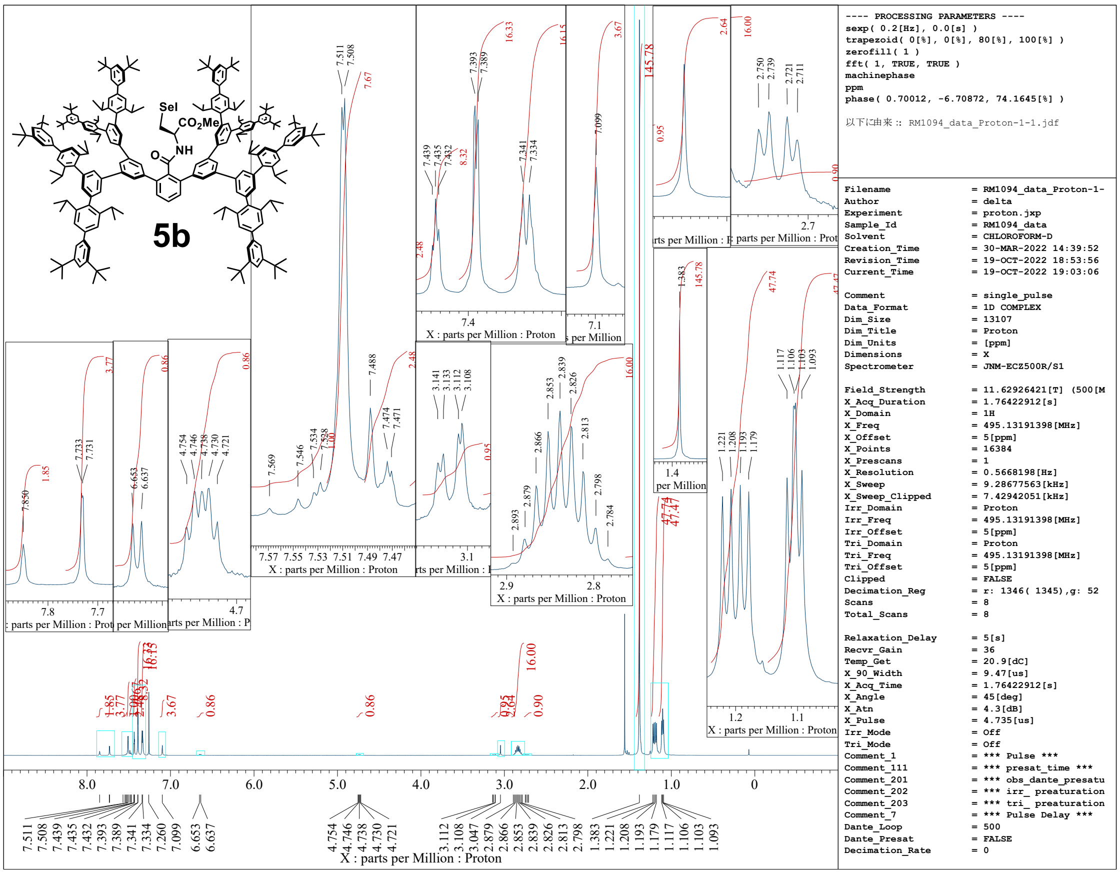

Figure S24. ^{1}H NMR (500 MHz, $CDCl_3$) spectrum of **5b**.

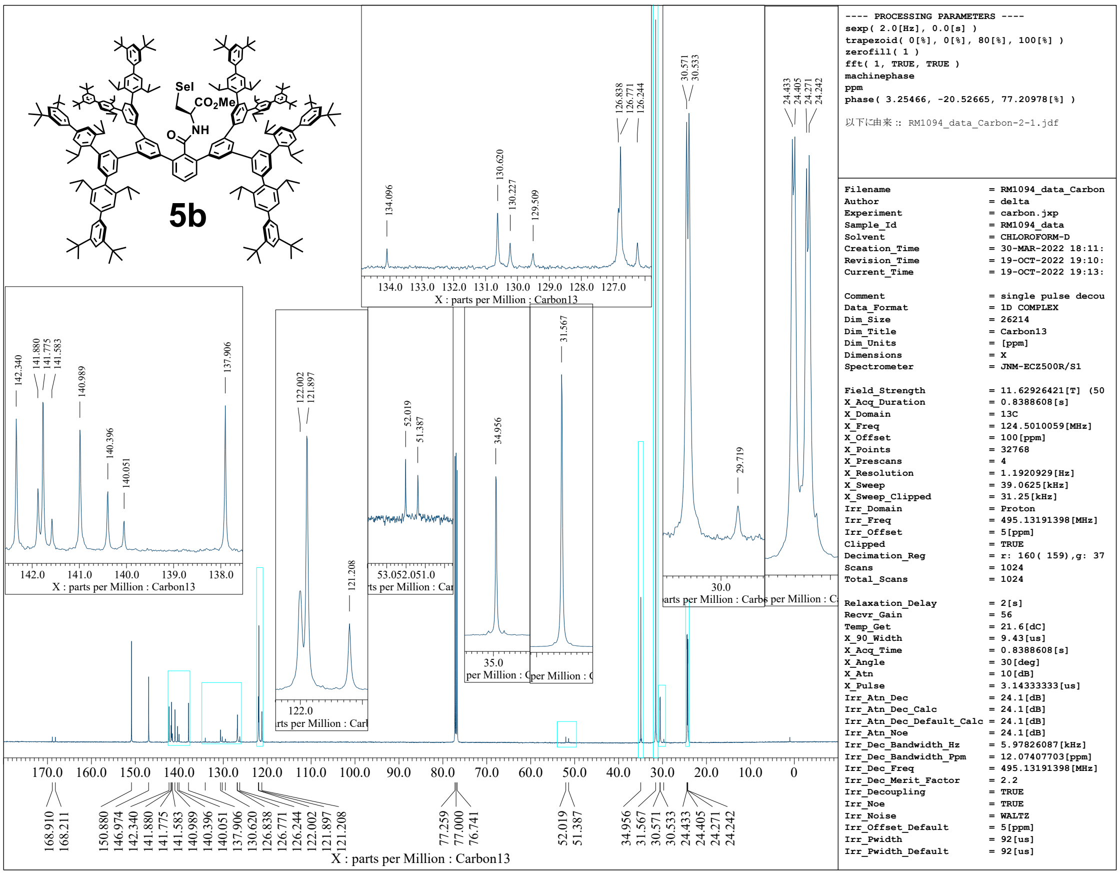

Figure S25. ^{13}C NMR (125 MHz, $CDCl_3$) spectrum of **5b**.

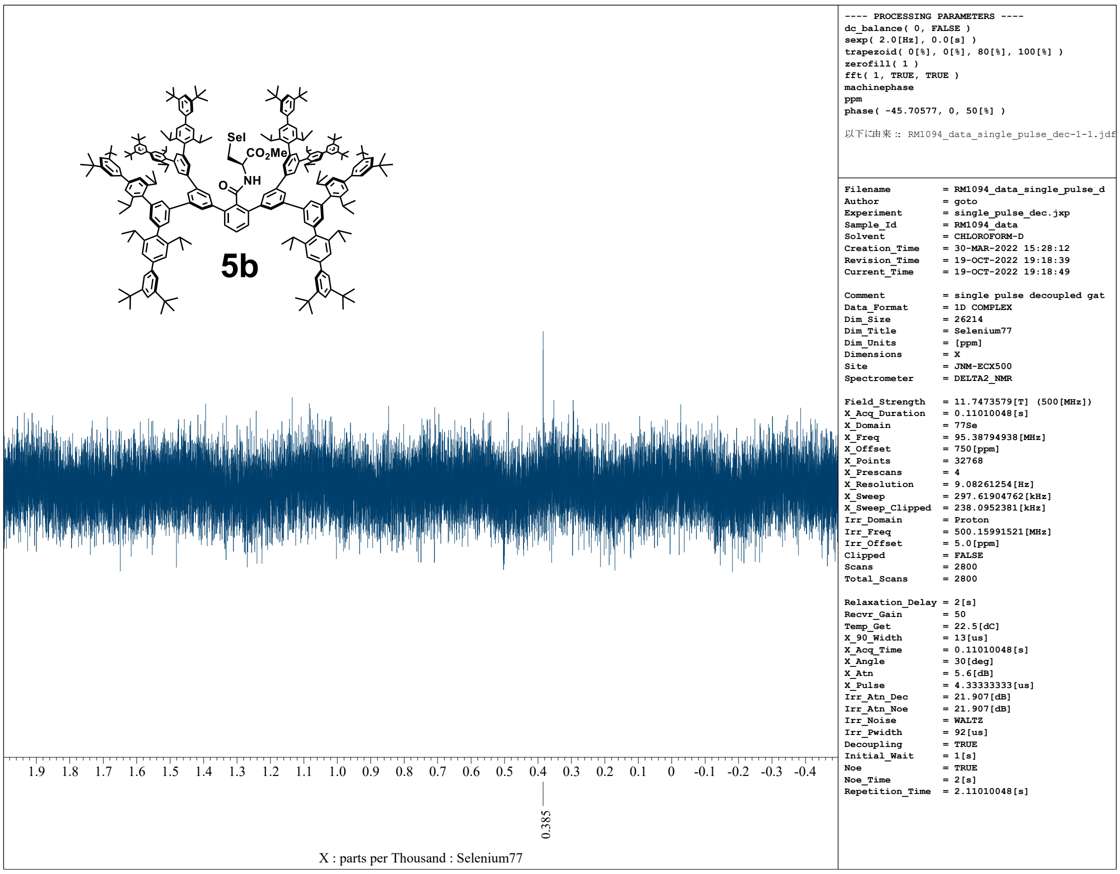

Figure S26. ^{77}Se NMR (95 MHz, $CDCl_3$) spectrum of **5b**.

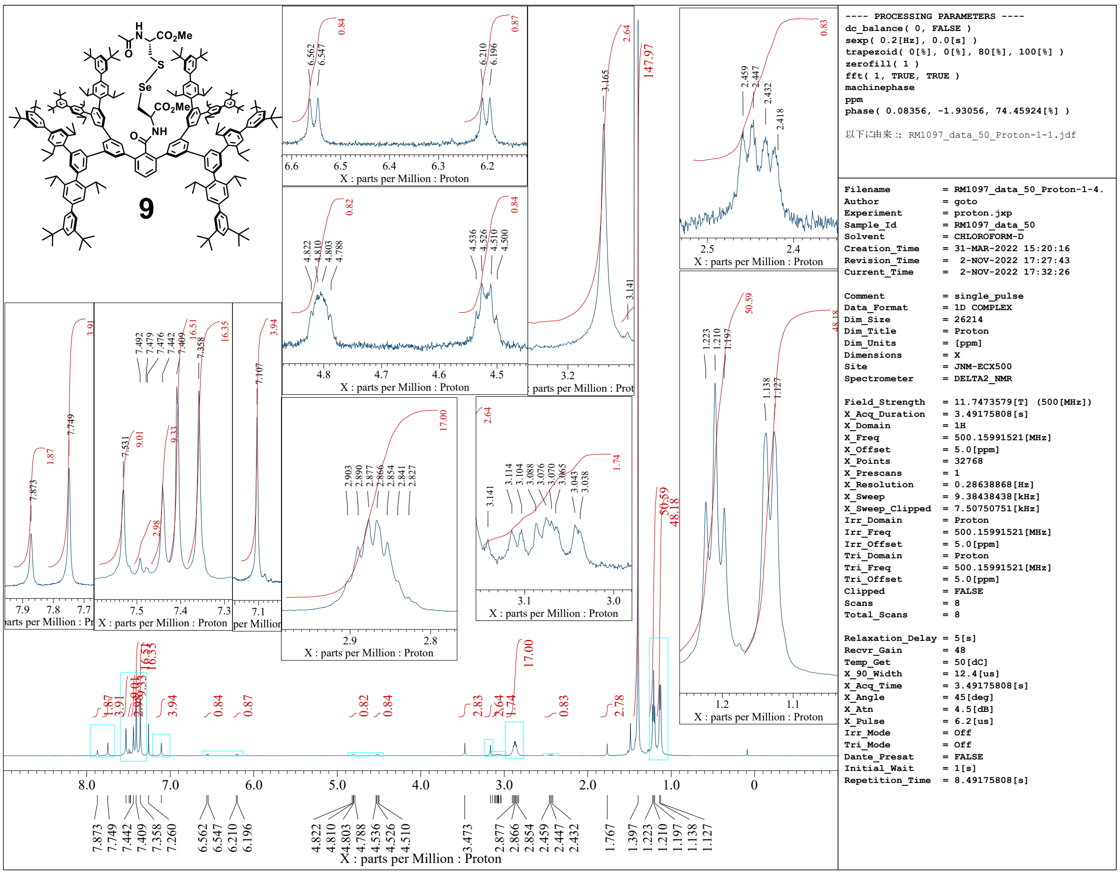

Figure S27. ^{1}H NMR (500 MHz, $CDCl_3$, 50 °C) spectrum of **9**.

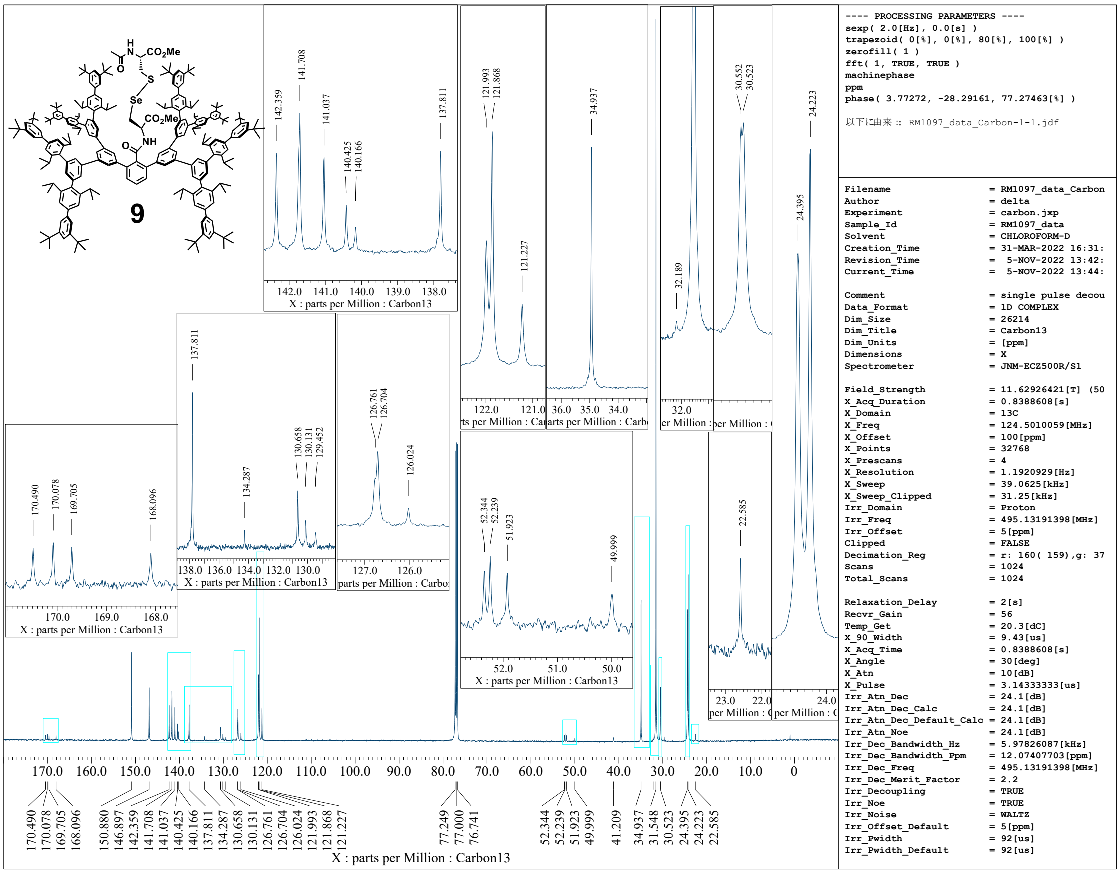

Figure S28. ^{13}C NMR (125 MHz, $CDCl_3$) spectrum of **9**.

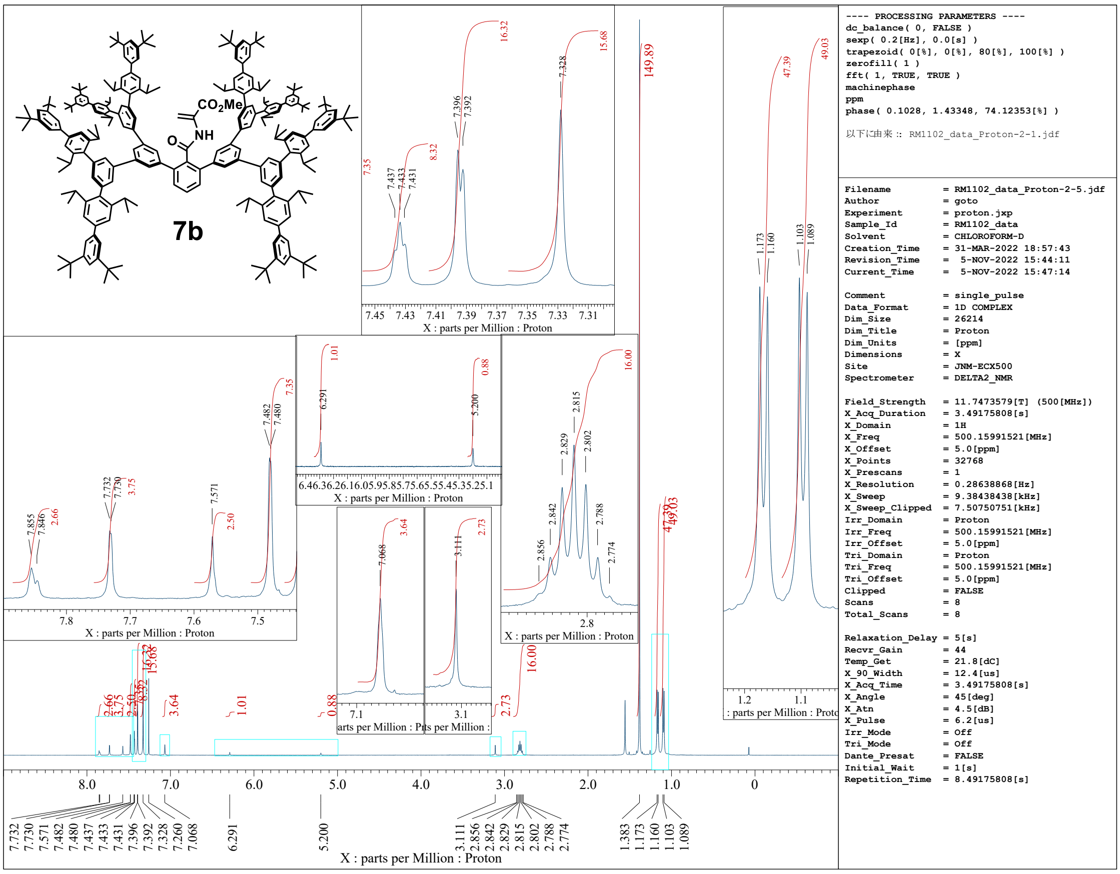

Figure S29. ^{1}H NMR (500 MHz, $CDCl_3$) spectrum of **7b**.

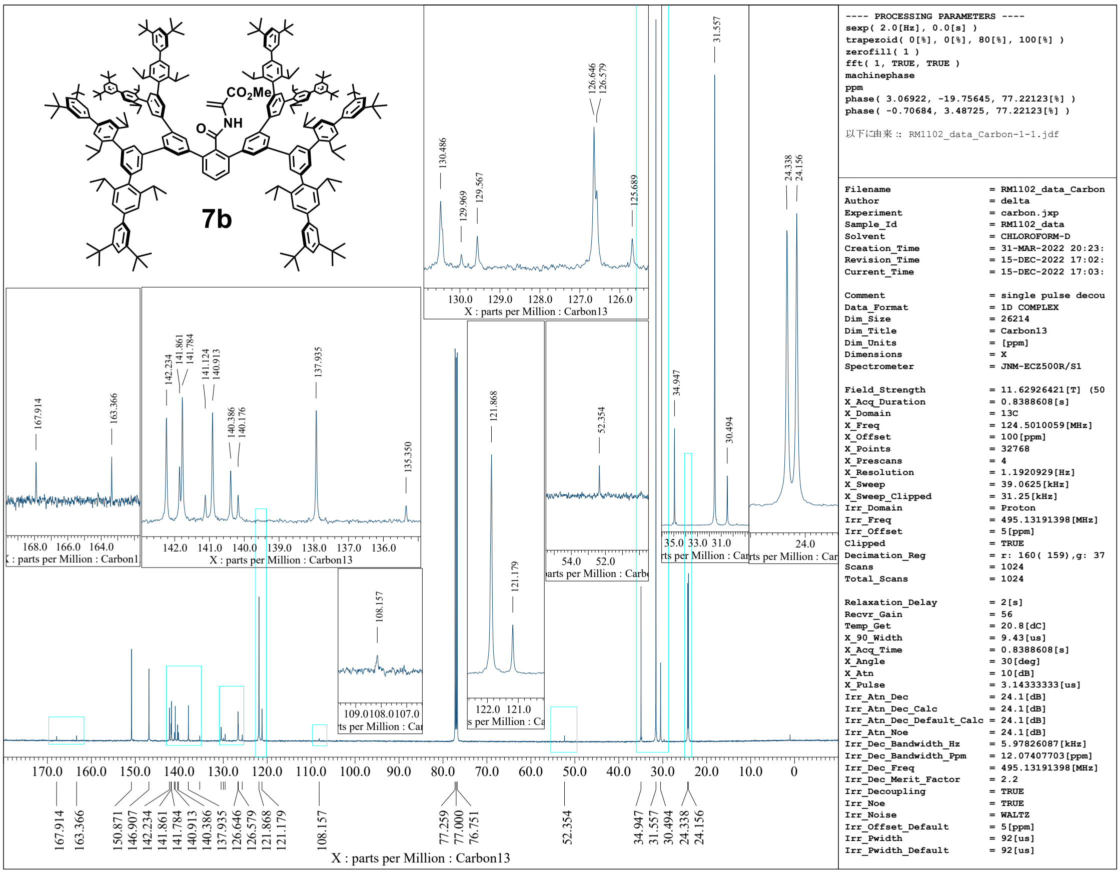

Figure S30. ^{13}C NMR (125 MHz, $CDCl_3$) spectrum of **7b**.

4. References

[1] (a) Masuda, R.; Kuwano, S.; Goto, K. Late-Stage Functionalization of the Periphery of Oligophenylene Dendrimers with Various Arene Units via Fourfold C-H Borylation. *J. Org. Chem.* **2021**, *86*, 14433–14443. (b) Masuda, R.; Goto, K. Modeling of Selenocysteine-Derived Reactive Intermediates Utilizing a Nano-Sized Molecular Cavity as a Protective Cradle, *Meth. Enzymol.* **2022**, *662*, 331–361.

[2] Sase, S.; Kimura, R.; Masuda, R.; Goto, K. Model Study on Trapping of Protein Selenenic Acids by Utilizing a Stable Synthetic Congener. *New J. Chem.* **2019**, *43*, 6830–6833.

[3] Stocking, E. M.; Schwarz, J. N.; Senn, H.; Silks, L. A. Synthesis of L-selenocystine, L-[^{77}Se]selenocystine and L-tellurocystine. *J. Chem. Soc. Perkin Trans. 1* **1997**, 2443–2447.

[4] Masuda, R.; Kimura, R.; Karasaki, T.; Sase, S.; Goto, K. Modeling the Catalytic Cycle of Glutathione Peroxidase by Nuclear Magnetic Resonance Spectroscopic Analysis of Selenocysteine Selenenic Acids. *J. Am. Chem. Soc.* **2021**, *143*, 6345–6350.

[5] Galloway, J. D.; Sarabia, C.; Fettinger, J. C.; Hratchian, H. P.; Baxter, R. D. Versatile New Reagent for Nitrosation. under Mild Conditions. *Org. Lett.* **2021**, *23*, 3253–3258.

[6] Shimada, K.; Goto, K.; Kawashima, T.; Takagi, N.; Choe, Y. K.; Nagase, S. Isolation of a *Se*-Nitrososelenol: A New Class of Reactive Nitrogen Species Relevant to Protein *Se*-Nitrosation. *J. Am. Chem. Soc.* **2004**, *126*, 13238–13239.

[7] (a) Shimada, K.; Goto, K.; Kawashima, T. Thermolysis and Photolysis of Stable *Se*-Nitrososelenols. *Chem. Lett.* **2005**, *34*, 654–655. (b) Goto, K.; Shimada, K.; Kawashima, T. Syntheses of the First *Se*-Nitrososelenol and Related Compounds. *Phosphorus, Sulfur Silicon Relat. Elem.* **2005**, *180*, 945–949.

[8] Song, L.; Keul, F.; Mardyukov, A. Preparation and Spectroscopic Identification of Methyl-*Se*-Nitrososelenol. *Chem. Commun.* **2019**, *55*, 9943–9946.

[9] Lutzke, A.; Melvin, A. C.; Neufeld, M. J.; Allison, C. L.; Reynolds, M. M. Nitric Oxide Generation from *S*-Nitrosoglutathione: New Activity of Indium and a Survey of Metal Ion Effects. *Nitric Oxide* **2019**, *84*, 16–21.
